# Chemometric Organization and Structure–Property Relationships in an Industrial Polypropylene Product Portfolio

**DOI:** 10.3390/polym18162009

**Published:** 2026-08-18

**Authors:** Joaquín Hernández-Fernández, Juan Lopez-Martinez, Jhojan Salcedo-Castellar

**Affiliations:** 1 Chemistry Program, Department of Natural and Exact Sciences, University of Cartagena, San Pablo Campus, Cartagena de Indias 130015, Colombia; jsalcedoc@unicartagena.edu.co; 2 Department of Natural and Exact Science, Universidad de la Costa, Barranquilla 080002, Colombia; 3 Grupo de Investigación GIA, Fundacion Universitaria Tecnologico Comfenalco, Cr 44 D N 30A, 91, Cartagena 130015, Colombia; 4 Institute of Materials Technology (ITM), Universitat Politecnica de Valencia (UPV), Plaza Ferrandiz and Carbonell s/n, 03801 Alcoy, Spain; jlopezm@mcm.upv.es

**Keywords:** polypropylene, industrial portfolio, structure–property relationships, chemometrics, principal component analysis, partial least squares discriminant analysis, family-level discrimination, heterophasic copolymers, polymer informatics, portfolio organization

## Abstract

Industrial polypropylene portfolios comprise multiple commercial grades differentiated by molecular architecture, phase morphology, processability, and performance. In this study, 81 industrial polypropylene grades, including 38 homopolymers, 21 random copolymers, and 22 impact copolymers, were analyzed to evaluate the chemometric organization and structure–property relationships of a complete commercial portfolio. The dataset integrated melt flow index, xylene-soluble fraction, total ethylene content, ethylene content of the rubber phase, rubber-phase fraction, and mechanical, thermal, and optical performance variables obtained from routine industrial quality-control and product-certification activities. Principal component analysis, partial least squares discriminant analysis, and variable importance in projection analysis were used to examine portfolio organization, evaluate consistency with the predefined polypropylene families, and identify the descriptors contributing most strongly to family-level discrimination. The first two principal components explained 83.8% of the total variance. They revealed a low-dimensional organization consistent with the molecular and morphological differences among homopolymer, random copolymer, and impact copolymer grades. The full-descriptor PLS-DA model achieved 98.8% cross-validated accuracy and correctly classified 80 of the 81 grades using two latent variables. This performance reflects the internal consistency between the descriptor matrix and the existing industrial family classification rather than independently validated predictive capability for unknown grades. Homopolymer differentiation was mainly associated with molecular-weight-related flow behavior, random copolymer organization with ethylene-induced modification of crystallinity, and impact copolymer differentiation with heterophasic rubber-phase characteristics. The results provide a portfolio-specific chemometric workflow for grade organization and structure–property interpretation. However, the numerical domain boundaries and their transferability require validation using independent polypropylene portfolios from other producers.

## 1. Introduction

Polypropylene is one of the most versatile and commercially relevant thermoplastics in the global polyolefins industry because of its favorable balance of cost, processability, chemical resistance, low density, and mechanical performance [1,2]. Its broad application range arises from the ability to tailor chain architecture, stereoregularity, comonomer incorporation, molecular weight, and multiphase morphology through catalyst selection and industrial polymerization design. Consequently, polypropylene grades can be engineered for applications including raffia, fibers, films, thermoformed packaging, injection-molded components, household appliances, and high-impact structural parts used in automotive applications [3,4]. This versatility, however, also introduces substantial complexity because final performance is not governed by a single molecular parameter but by the coupled effects of molecular architecture, crystallinity, phase morphology, melt processability, thermal response, optical behavior, and application-specific requirements [2,5].

The structure–property relationships of polypropylene are intrinsically hierarchical. In homopolymers, molecular weight and molecular-weight distribution strongly influence melt flow, stiffness, crystallinity, melt strength, and processing behavior [6,7]. Melt flow index (MFI) is widely used in industrial practice as a rapid descriptor of processability and as an indirect indicator of molecular-weight-related flow behavior. Nevertheless, MFI does not fully describe the viscoelastic response of polypropylene melts, because shear thinning, melt elasticity, elongational viscosity, and the effects of molecular-weight distribution are not captured by a single extrusion-based flow value [8]. In random copolymers, ethylene incorporation disrupts the regularity of isotactic propylene sequences, reducing crystalline perfection and enabling adjustments in transparency, ductility, impact response, and stiffness [9,10]. In impact copolymers, the material architecture becomes more complex because a semicrystalline polypropylene matrix coexists with a dispersed ethylene–propylene elastomeric phase. In these systems, rubber-phase ethylene content (Rubber EC) and rubber-phase fraction (Rubber FC) are key descriptors governing toughness, energy dissipation, and low-temperature impact resistance [11,12].

Processing history further contributes to the complexity of polypropylene performance.

Industrial grades are selected not only according to molecular architecture and composition, but also according to their suitability for specific processing routes, including extrusion, thermoforming, injection molding, fiber spinning, film production, and high-productivity molding operations. In this context, MFI is widely used as a practical industrial descriptor of molecular-weight-related processability. However, it does not fully resolve shear-thinning behavior, melt elasticity, elongational viscosity, relaxation dynamics, or molecular-weight-distribution effects.

Although the molecular principles governing individual polypropylene grades are well established, their integration across complete industrial product portfolios remains insufficiently resolved. Most structure–property studies focus on individual grades, selected formulations, binary blends, specific catalyst systems, or isolated polypropylene families. Other studies use polymer informatics or machine-learning approaches for property prediction [13,14,15,16,17,18,19,20]. However, they often rely on literature-derived datasets, simulated descriptors, or material classes that are not organized as complete commercial portfolios. As a result, three important gaps remain. First, grade-level industrial datasets that combine molecular, compositional, mechanical, thermal, optical, and application-related descriptors are rarely analyzed as complete portfolio systems. Second, the organization of homopolymer, random copolymer, and impact copolymer grades within a unified molecular design space is not commonly quantified. Third, the extent to which chemometric methods can support the interpretation of predefined industrial families and product domains remains underexplored.

Commercial polypropylene portfolios are not arbitrary collections of isolated products. They are structured design spaces in which molecular architecture, phase morphology, processability, performance balance, and commercial application windows must be simultaneously controlled. This creates a multivariate problem: two grades may differ only slightly in a single descriptor, such as MFI or total ethylene content, yet occupy different application spaces due to combined differences in stiffness, toughness, optical response, thermal resistance, and morphology. Conventional univariate analysis is therefore insufficient to resolve the latent structure of such portfolios, particularly when descriptors are correlated or when multiple performance targets are optimized simultaneously [12,13].

Chemometric and multivariate methods provide a suitable framework to address this type of complexity. Principal component analysis (PCA) enables dimensionality reduction and visualization of latent structure within correlated polymer datasets [21,22]. Partial least squares discriminant analysis (PLS-DA) allows supervised discrimination among predefined material families or product groups [23,24,25]. Variable importance in projection (VIP) analysis can then be used to identify which descriptors contribute most strongly to the supervised discrimination structure [26,27]. When combined with correlation analysis and domain-level standardized profiles, these tools can transform complex industrial datasets into chemically interpretable structure–property–application maps. However, their application to the organization of complete industrial polypropylene portfolios remains limited.

In this context, the present work investigates an industrial portfolio comprising 81 commercial polypropylene grades, including 38 homopolymers, 21 random copolymers, and 22 impact copolymers. The dataset integrates molecular and compositional descriptors, including MFI, xylene-soluble fraction, total ethylene content, ethylene content of the rubber phase, and rubber-phase fraction, together with mechanical, thermal, and optical performance variables obtained from routine industrial quality-control and product-certification activities. PCA, PLS-DA, VIP analysis, Pearson correlation analysis, and standardized domain-level profiling were applied to evaluate the latent organization of the descriptor matrix, its consistency with the predefined industrial polypropylene families, and the variables contributing to portfolio differentiation.

The objective of this study was to establish a portfolio-specific chemometric workflow for organizing commercial polypropylene grades and interpreting structure–property relationships within an industrial dataset. The nine product domains evaluated in this work—low-, medium-, and high-flow homopolymers; low-, medium-, and high-ethylene random copolymers; and standard-impact, high-impact, and high-flow impact copolymers—were defined before chemometric modeling using industrial product specifications, molecular architecture, compositional characteristics, rheological behavior, performance profile, and commercial positioning. Therefore, the multivariate methods were not used to generate application categories or universal polypropylene classifications. Instead, they were used to assess whether the measured descriptor matrix was consistent with the predefined family and domain organization. Because all grades were obtained from a single industrial portfolio, the numerical domain boundaries and population structure reported here should be interpreted as portfolio-specific. The study’s transferable contribution lies in the chemometric workflow that integrates molecular, compositional, and performance information to support portfolio organization and structure–property interpretation.

## 2. Materials and Methods

ASTM standards are cited in this section by their corresponding designations because they were used exclusively as standardized testing protocols for industrial quality-control measurements. They are not included in the reference list because they do not constitute part of the scientific literature used to support the structure–property interpretation of the polypropylene portfolio.

### 2.1. Polypropylene Samples and Industrial Portfolio

This study was conducted using an industrial polypropylene portfolio comprising 81 commercial grades manufactured by Esenttia S.A. under continuous industrial production conditions. The portfolio included 38 homopolymers, 21 random copolymers, and 22 impact copolymers, covering a broad range of commercial applications, including raffia, fibers, films, thermoforming, injection molding, household appliances, packaging, and automotive components.

The study was designed to evaluate the complete commercial portfolio available during the data collection period rather than a statistically selected subset. Consequently, the dataset captures the full diversity of molecular architectures, compositional characteristics, rheological behaviors, mechanical performance levels, thermal responses, optical properties, and application spaces represented within the industrial portfolio. All data were obtained from routine industrial quality control, product certification, process monitoring, and product-development activities. Therefore, the dataset reflects the actual characteristics of commercially manufactured polypropylene grades rather than simulated values or literature-derived estimations.

The homopolymer family comprised 38 commercial grades developed primarily for raffia, fiber, extrusion, thermoforming, and injection molding applications. These grades cover a broad melt-flow range and were used to evaluate the role of molecular-weight-related processability in portfolio differentiation. The random copolymer family comprised 21 grades designed mainly for packaging, films, sheets, rigid containers, thermoforming, and transparent food-contact applications. These materials were used to assess the effect of ethylene incorporation on crystallinity, optical performance, and mechanical behavior. The impact copolymer family comprised 22 heterophasic grades intended for appliances, industrial products, packaging, and automotive applications. Their inclusion enabled the evaluation of the influence of the rubber phase fraction and the total ethylene content of the elastomeric phase on the performance of impact polypropylene materials.

For FTIR-based compositional characterization, representative samples from each grade were prepared as compression-molded films using the standard procedures employed by the industrial quality-control laboratory. This procedure was used to ensure adequate spectral reproducibility and thickness uniformity prior to determining the total ethylene content, the total ethylene content in the rubber phase, and the Rubber EC.

### 2.2. Melt Flow Index Determination

Melt flow index measurements were performed according to ASTM D1238-23a [28] under standard polypropylene testing conditions of 230 °C and a 2.16 kg load. MFI values were reported in g/10 min and used as an indirect descriptor of molecular weight, chain mobility, melt rheology, and processing behavior. Because MFI is inversely related to molecular weight, this descriptor provides relevant information for differentiating grades designed for high melt strength, balanced processability, or high-flow applications.

### 2.3. Determination of Xylene-Soluble Fraction

The xylene-soluble fraction was determined using standard industrial procedures based on selective dissolution of the amorphous polymer fraction in boiling xylene, followed by gravimetric quantification of the soluble material. XS values were expressed as weight percentages and used as descriptors of amorphous content, crystallinity reduction, and morphological heterogeneity. This parameter is particularly relevant for distinguishing polypropylene grades modified by comonomer incorporation or elastomeric-phase development.

### 2.4. Determination of Total Ethylene Content and Rubber EC

Total ethylene content was determined by Fourier-transform infrared spectroscopy using a Thermo Scientific Nicolet iS10 spectrometer (Thermo Fisher Scientific, Madison, WI, USA) equipped with a diamond attenuated total reflectance accessory. Compression-molded films representative of the commercial grades were analyzed over the 4000–400 cm^−1^ spectral range using a resolution of 4 cm^−1^ and 32 accumulated scans per spectrum.

Total ethylene content was quantified using the industrial calibration methodology routinely employed for polypropylene product certification and quality control. Results were expressed as weight percentages and used to describe the overall comonomer incorporation in random and impact copolymers. Because ethylene units disrupt the regularity of isotactic propylene sequences, total ethylene content is associated with changes in crystallization behavior, crystalline morphology, optical response, ductility, and stiffness.

For impact copolymers, the ethylene content of the elastomeric rubber phase, hereafter denoted Rubber EC, was determined using the same FTIR-ATR platform, film-preparation procedure, and spectral acquisition conditions. Rubber EC values were obtained from the industrial calibration model established for heterophasic polypropylene characterization and were expressed as a weight percentage. This descriptor characterizes the composition of the elastomeric phase and contributes to the interpretation of toughness, low-temperature impact response, and energy dissipation.

### 2.5. Determination of Rubber FC

The rubber-phase fraction, hereafter denoted Rubber FC, was determined by FTIR-ATR using the Thermo Scientific Nicolet iS10 spectrometer and the compression-molded film protocol described in Section 2.4. Rubber FC values were obtained using the routine industrial calibration methodology employed for impact-polypropylene quality certification and were expressed as a weight percentage.

Rubber EC and Rubber FC describe complementary characteristics of heterophasic impact copolymers. Rubber EC represents the ethylene content of the elastomeric phase, whereas Rubber FC represents the relative fraction of that phase within the material. Together, these descriptors characterize the composition and amount of the rubber phase responsible for the toughness and impact performance of heterophasic polypropylene grades.

The internal industrial procedures used to determine XS, total ethylene content, Rubber EC, and Rubber FC were applied consistently across the corresponding commercial grades. XS was obtained by selective dissolution of the xylene-soluble fraction followed by gravimetric quantification. Total ethylene content, Rubber EC, and Rubber FC were obtained from FTIR-ATR spectra acquired from compression-molded films with controlled thickness and surface uniformity. Quantification was performed using industrial calibration models previously established for polypropylene quality certification. Although the proprietary calibration equations cannot be disclosed, the same sample-preparation protocol, acquisition conditions, calibration framework, and quality-control criteria were applied to all grades belonging to the relevant material family. These procedures ensured the internal consistency of the compositional and phase-composition descriptors included in the chemometric matrix.

### 2.6. Mechanical, Thermal, and Optical Characterization

Mechanical, thermal, and optical properties were measured using standardized procedures routinely employed for industrial polypropylene qualification. Tensile properties were determined according to ASTM D638 [29], and flexural modulus was measured according to ASTM D790 [30]. Impact resistance was evaluated using the notched Izod impact test in accordance with ASTM D256 [31]. Thermal performance was assessed using Vicat softening temperature measurements per ASTM D1525 [32] and heat deflection temperature measurements per ASTM D648 [33]. Optical performance was evaluated using haze measurements in accordance with ASTM D1003 [34], when applicable. These descriptors collectively characterize stiffness, strength, toughness, thermal resistance, and optical behavior across the industrial portfolio.

### 2.7. Industrial Portfolio Database

The complete dataset was integrated into a unified structure–property database. Table 1 summarizes the composition of the industrial polypropylene portfolio and the relative representation of the three principal molecular architectures.

To improve data transparency and reproducibility, the grade-level data matrix used for the chemometric workflow is provided as a separate Excel file in the Supplementary Materials (Supplementary Table S9). Commercial-grade names were replaced by sequential codes (PP-001 to PP-081), while preserving the family labels, product-domain labels, and the descriptors used in PCA, PLS-DA, VIP analysis, Pearson correlation analysis, and domain-level profiling. Confidential information related to grade identities, production recipes, catalyst details, process set points, customer-specific specifications, and proprietary product-development criteria was not disclosed. A data-status field is included in the Supplementary Dataset to document the provenance or verification status of each record; this field was used only for transparency and was not included as a modeling variable.

### 2.8. Molecular, Compositional, and Performance Descriptors

The descriptors included in the chemometric analysis were grouped into molecular/processability, compositional, phase-composition, and performance categories, as summarized in Table 2. MFI was treated as a molecular-weight-related processability proxy, whereas XS and total ethylene content were treated as compositional or morphological descriptors. Rubber EC and Rubber FC were classified as phase-composition descriptors that are physically meaningful only for heterophasic impact copolymers. Rubber EC denotes the ethylene content of the elastomeric rubber phase, whereas Rubber FC denotes the relative fraction of that phase. Performance descriptors included tensile strength, tensile elongation, flexural modulus, notched Izod impact, Vicat softening temperature, heat deflection temperature (HDT), and haze. This terminology distinguishes molecular, compositional, and phase-related variables from the mechanical, thermal, and optical responses resulting from polypropylene architecture and morphology.

A complete summary of the analytical techniques, instrumentation, and standard methods used for portfolio characterization is provided in the Supplementary Information.

### 2.9. Structure–Property Database and Product-Domain Definition

The structure–property database was constructed by integrating MFI, XS, total ethylene content, Rubber EC, and Rubber FC with the mechanical, thermal, and optical performance variables obtained during routine industrial characterization.

Product domains were defined prior to chemometric modeling based on established industrial product specifications, commercial positioning, molecular architecture, rheological behavior, comonomer incorporation, elastomeric-phase characteristics, performance profile, and intended application space. Therefore, PCA and PLS-DA were not used to create the domains a posteriori. Instead, these methods were used to evaluate whether the experimental descriptor matrix supported a coherent chemometric organization of the predefined industrial domains. Application categories were treated as contextual industrial metadata associated with the predefined domains. They were not included as response variables or outcomes derived from PCA, PLS-DA, VIP, or correlation analysis. Consequently, the chemometric results were interpreted in terms of portfolio organization and structure–property consistency rather than independent application prediction.

The following domain abbreviations were used throughout the manuscript: H-LF, low-flow homopolymer; H-MF, medium-flow homopolymer; H-HF, high-flow homopolymer; R-LE, low-ethylene random copolymer; R-ME, medium-ethylene random copolymer; R-HE, high-ethylene random copolymer; C-STD, standard-impact copolymer; C-HI, high-impact copolymer; and C-HMF, high-flow impact copolymer.

The domain-assignment criteria are summarized in Table 3. These criteria should be interpreted as portfolio-specific operational rules rather than universal polypropylene thresholds. The purpose of the classification was to preserve the industrial meaning of each grade while enabling chemometric evaluation of the molecular, compositional, and performance gradients that organize the portfolio.

### 2.10. Chemometric Analysis

#### 2.10.1. Data Preprocessing and Multivariate Dataset Construction

Before multivariate analysis, all variables were examined for consistency, completeness, and numerical compatibility. The final data matrix included molecular descriptors, phase-composition variables, mechanical properties, thermal properties, and optical descriptors. Because these variables have different units and numerical ranges, all X variables were mean-centered and autoscaled prior to chemometric modeling. This preprocessing step ensured that the multivariate models were driven by correlation structure rather than by differences in measurement scale [35].

Special attention was given to descriptors that are structurally absent in specific polypropylene families. Rubber EC and Rubber FC describe, respectively, the ethylene content and relative fraction of the elastomeric phase in heterophasic impact copolymers. These variables are therefore physically meaningful for impact copolymers but structurally absent in homopolymers and random copolymers. In the unified chemometric matrix, Rubber EC and Rubber FC were coded as structural zeros for homopolymer and random copolymer grades rather than as missing values or imputed estimates. Similarly, total ethylene content was coded as zero for homopolymer grades because these materials contain no comonomer incorporation. These zero values represent the absence of the corresponding structural feature and not an unmeasured analytical result. Missing-value handling was limited to three molecular-descriptor entries in the R-LE domain. These values were imputed using the median of the corresponding descriptor within the same product domain to preserve the full 81-grade matrix for cross-validation. No family labels, domain labels, or performance descriptors were used for this imputation. This preprocessing decision was necessary to construct a unified portfolio-level matrix containing homopolymers, random copolymers, and impact copolymers. However, it also has an important interpretive consequence: part of the separation observed in PCA, PLS-DA, VIP analysis, and Pearson correlation analysis may reflect family-level structural absence or presence of comonomer and rubber-phase descriptors. For this reason, the multivariate analyses were interpreted as portfolio-level discrimination and structure–property organization, not as universal within-family structure–property laws. The role of structural-zero descriptors was considered in discussions of the loading plot, VIP scores, and correlation heatmap.

All numerical variables included in the X matrix were mean-centered and autoscaled before PCA and PLS-DA. Identifier columns, commercial-code replacements, family labels, product-domain labels, and metadata fields included in Supplementary Table S9 were not used as X variables. The autoscaled matrix was used to evaluate the latent organization of the portfolio. In contrast, the family and domain labels were used only for visualization, interpretation, or supervised class assignment, as appropriate.

All chemometric analyses were performed using OriginPro 2024. The resulting autoscaled matrix represented the multidimensional structure of the industrial polypropylene portfolio. It served as the basis for PCA, PLS-DA, VIP analysis, product-domain interpretation, and structure–property correlation analysis.

For model construction, only the numerical descriptor columns from Supplementary Table S9 were included in the X matrix. Coded grade identifiers, product-domain labels, and the data-status field were excluded from the predictor matrix. Family labels were used only to construct the dummy Y matrix required for PLS-DA, whereas domain labels were used for domain-level interpretation and application mapping.

#### 2.10.2. Principal Component Analysis

Principal component analysis was used as an unsupervised multivariate method to explore the intrinsic organization of the polypropylene portfolio [21,22]. PCA transforms correlated experimental descriptors into orthogonal latent variables that maximize explained variance. This approach enables visualization of clustering behavior, detection of molecular domains, evaluation of relationships among descriptors, and identification of the dominant sources of portfolio variability.

Score plots were used to evaluate similarities and differences among commercial grades, whereas loading plots were used to determine the contribution of each descriptor to portfolio differentiation. The percentage of variance explained by each principal component was used to assess the efficiency of dimensionality reduction and the representativeness of the selected components.

#### 2.10.3. Partial Least Squares Discriminant Analysis

Partial least squares discriminant analysis (PLS-DA) was applied as a supervised method to evaluate discrimination among the three principal polypropylene families: homopolymers, random copolymers, and impact copolymers [36,37,38,39]. Family membership was encoded using a dummy response matrix with one binary column per family. The class assignment was performed based on the highest predicted class value.

Two PLS-DA models were evaluated to distinguish the contribution of molecular/compositional descriptors from that of the complete portfolio descriptor matrix. The first model, referred to as the molecular/compositional PLS-DA model, included MFI, XS, total ethylene content, Rubber EC, and Rubber FC. The second model, referred to as the full-descriptor PLS-DA model, included these variables together with tensile strength, tensile elongation, heat deflection temperature, Vicat softening temperature, and haze. This comparison was performed to evaluate whether family-level discrimination was driven primarily by molecular and phase-composition descriptors or further strengthened by downstream performance variables.

Three missing molecular-descriptor entries were detected in the grade-level matrix and were imputed using the median value of the corresponding product domain. Specifically, one MFI value and two XS values in the R-LE domain were imputed using the R-LE domain median. All variables were mean-centered and autoscaled prior to modeling.

The number of latent variables was fixed at two for both models to avoid overparameterization and to maintain direct comparability between the molecular/compositional and full-descriptor models. Model performance was evaluated using calibration accuracy, stratified 5-fold cross-validation, precision, recall, F1-score, and confusion matrices. To assess whether the observed discrimination could arise from random class assignment, a permutation test was performed using 500 random permutations of the family labels.

PLS-DA was used as an internal supervised discrimination method to evaluate the consistency between the experimental descriptor matrix and the predefined industrial polypropylene families. It was not intended or validated as a predictive classifier for unknown grades or independent commercial portfolios. This distinction is particularly important because total ethylene content, Rubber EC, and Rubber FC are structurally associated with the definitions of homopolymer, random copolymer, and impact copolymer families. Their inclusion therefore contributes directly to the supervised separation of the known classes. Accordingly, high classification accuracy was interpreted as evidence of agreement between the measured descriptors and the existing industrial classification rather than as independent predictive capability.

Stratified 5-fold cross-validation and permutation testing were used to evaluate the internal stability of the discrimination and the probability of obtaining comparable separation after random reassignment of the family labels. However, these procedures do not replace external validation using polypropylene grades from an independent producer. Consequently, the reported performance metrics should be restricted to the analyzed portfolio.

#### 2.10.4. Variable Importance in Projection Analysis

Variable importance in projection scores was calculated from the PLS-DA model to identify the descriptors contributing most strongly to supervised discrimination [26,27]. VIP analysis provides a quantitative estimate of each variable’s influence on the latent classification structure. Variables with VIP scores above 1.0 were considered the most relevant contributors to the classification model. Variables with scores close to 1.0 were interpreted as secondary but still informative descriptors, particularly when their molecular significance was consistent with the structure–property trends observed in PCA and correlation analyses. Because several variables included in the PLS-DA model are structurally associated with the predefined family labels, the VIP scores were interpreted as measures of contribution to internal family discrimination rather than as evidence of universal predictive importance. In particular, the relevance of Rubber EC and Rubber FC reflects both their physicochemical significance in heterophasic polypropylene and their structural absence in homopolymer and random copolymer grades.

#### 2.10.5. Correlation and Structure–Property Analysis

Pairwise relationships between molecular descriptors and performance variables were evaluated using Pearson correlation analysis. The resulting correlation matrix was used to identify direct structure–property associations, including stiffness–toughness trade-offs, relationships between elastomeric-phase descriptors and impact response, and links between crystallinity-related descriptors and optical behavior.

In addition, standardized domain-level means were calculated to compare molecular and performance profiles across the nine product domains. These standardized values were used to generate structure–property heatmaps, where positive values indicated domain means above the portfolio average and negative values indicated domain means below the portfolio average. This analysis enabled the transformation of statistical patterns into chemically interpretable molecular domains.

#### 2.10.6. Product-Domain Interpretation and Industrial Contextualization

The results obtained from PCA, PLS-DA, VIP analysis, Pearson correlation analysis, and domain-level standardized profiles were integrated to interpret the molecular, compositional, and performance characteristics of the predefined product domains. The chemometric analyses were not used to create the domains or derive application categories a posteriori. Instead, they were used to assess whether the experimental descriptor matrix supported the portfolio’s existing industrial organization.

Commercial application information was used only as contextual metadata to describe the industrial positioning of the analyzed grades. Therefore, the associations between product domains and application spaces should be interpreted as portfolio-specific industrial context rather than as application predictions generated by the chemometric models.

## 3. Results and Discussion

### 3.1. Composition and Hierarchical Organization of the Polypropylene Portfolio

**Figure 1:**
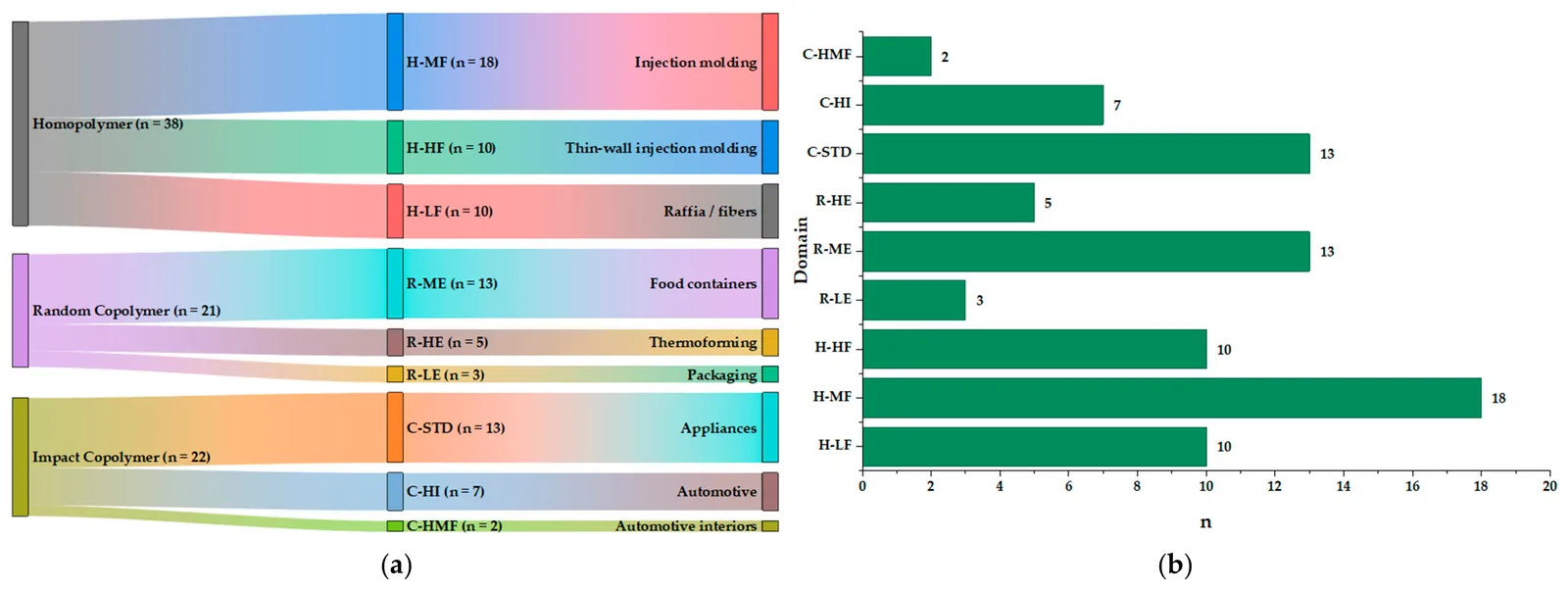
Hierarchical organization and domain population of the industrial polypropylene portfolio. (**a**) Predefined organization of the three polypropylene families, nine product domains, and their established industrial-use contexts. Flow width is proportional to the number of commercial grades assigned to each family, domain, and application category. Application information represents contextual industrial metadata and was not derived from the chemometric models. (**b**) Number of commercial grades assigned to each product domain. Domains are ordered according to the homopolymer, random copolymer, and impact copolymer families.

The industrial polypropylene portfolio was first evaluated as a hierarchical molecular and performance organization. Figure 1 integrates two complementary levels of information: the distribution of the 81 commercial grades according to their predefined family and product-domain classifications, and the number of grades represented in each domain. The application categories shown in the figure correspond to the established industrial positioning of the grades and are included for contextual interpretation; they were not generated independently by the chemometric analysis.

The first hierarchical level separates the portfolio into homopolymers, random copolymers, and impact copolymers, corresponding to progressively more complex polypropylene architectures. Homopolymers represent crystalline polypropylene matrices without comonomer or rubber-phase modification. Random copolymers introduce ethylene units into the polypropylene chain, altering crystalline regularity, optical response, and mechanical balance [9,10,37,38]. Impact copolymers exhibit a heterophasic morphology in which a polypropylene matrix coexists with a dispersed elastomeric phase, a structural feature directly related to toughness and impact energy dissipation [3,11,12,24].

The second hierarchical level separates each family into predefined product domains. Homopolymer domains mainly reflect melt-flow differentiation and molecular-weight-related processability. Random copolymer domains reflect increasing ethylene incorporation and its influence on crystalline organization, transparency, and ductility. Impact copolymer domains represent heterophasic materials differentiated by rubber-phase characteristics, impact performance, and melt-flow requirements. Figure 1 therefore summarizes the existing industrial organization of the analyzed portfolio and provides the contextual basis for evaluating whether the measured descriptors reproduce chemically interpretable family- and domain-level patterns.

The domain population profile indicates that the portfolio is concentrated in a limited number of high-utility domains. H-MF contains the highest number of grades, confirming that medium-flow homopolymers constitute the central homopolymer platform of the portfolio. R-ME and C-STD are also strongly represented, indicating that medium-ethylene random copolymers and standard-impact copolymers occupy major commercial regions. In contrast, R-LE and C-HMF are the least populated domains and should therefore be interpreted as specialized portfolio niches rather than statistically broad product spaces.

The product domains discussed below correspond to the portfolio-specific assignment criteria defined in Table 3. Therefore, the subsequent chemometric analysis should be interpreted as an evaluation of whether the measured descriptor matrix supports the predefined industrial domain structure, rather than as an unsupervised procedure used to create the domains.

Figure 1 shows that the portfolio follows a structured molecular design logic rather than a random commercial grouping of grades. The first hierarchical level separates the portfolio into homopolymers, random copolymers, and impact copolymers, which correspond to progressively more complex polypropylene architectures. Homopolymers represent crystalline polypropylene matrices without comonomer or rubber-phase modification. Random copolymers introduce ethylene units into the polypropylene chain, altering crystalline regularity, optical response, and mechanical balance [8,9,23]. Impact copolymers incorporate a heterophasic morphology in which a polypropylene matrix coexists with a dispersed elastomeric phase, a structural feature directly related to toughness and impact-energy dissipation [3,10,11,23,24].

The second hierarchical level divides each family into specific product domains. For homopolymers, the H-LF, H-MF, and H-HF domains primarily reflect melt-flow differentiation and, therefore, molecular-weight control. For random copolymers, R-LE, R-ME, and R-HE reflect increasing ethylene incorporation and its influence on crystallinity disruption, transparency, and ductility. For impact copolymers, C-STD, C-HI, and C-HMF represent heterophasic materials differentiated by Rubber EC, impact performance, and melt-flow requirements. Therefore, Figure 1 provides the first evidence that the portfolio is organized through a molecularly interpretable structure–property–application sequence.

**Figure 2:**
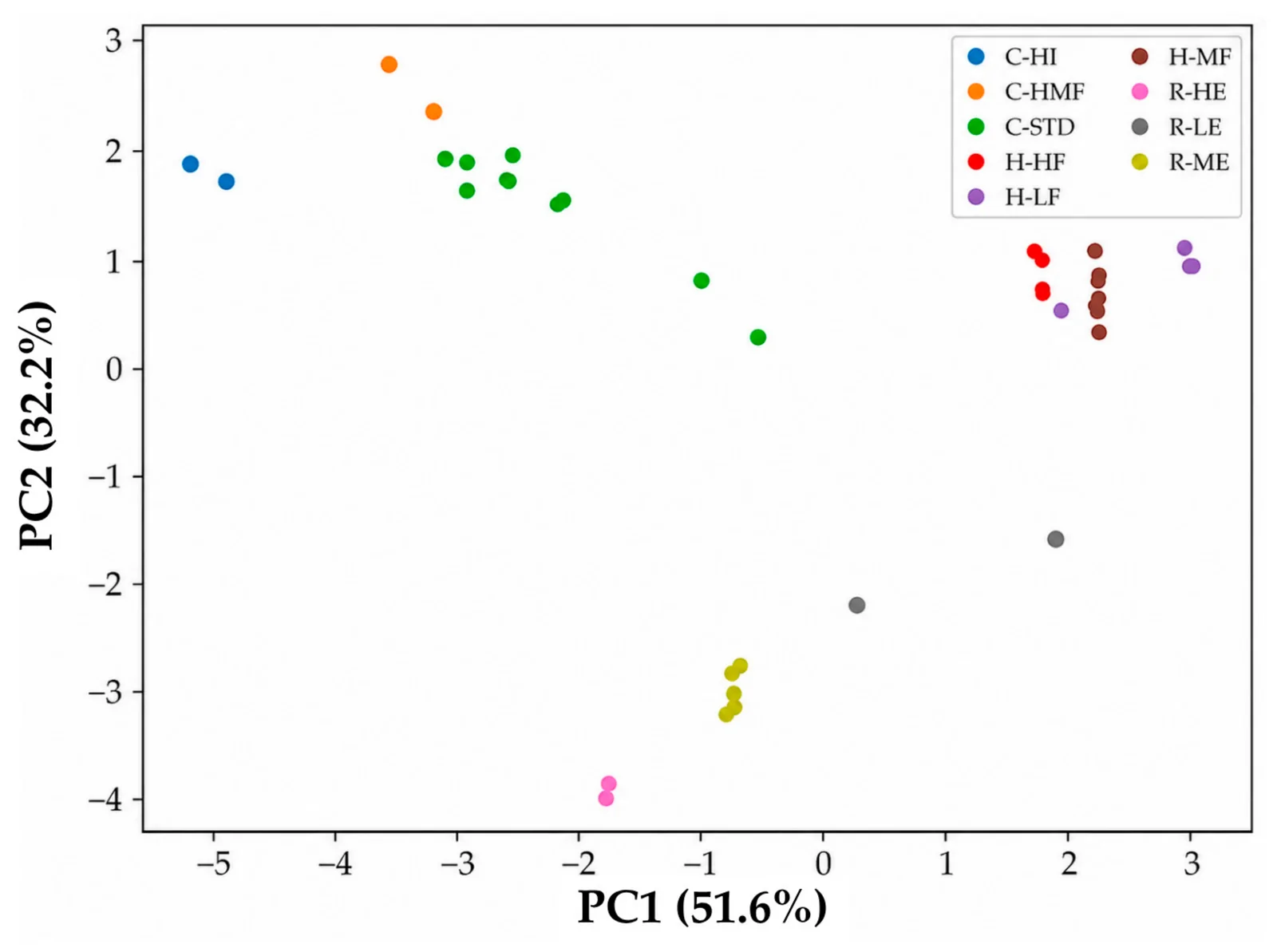
PCA score plot of the autoscaled structure–property matrix for the industrial polypropylene portfolio. PC1 and PC2 explain 51.6% and 32.2% of the total variance, respectively. Each point represents a commercial polypropylene grade and is colored by product domain. Domain labels were enlarged to improve readability.

Table 4 summarizes the mean molecular descriptors defining the nine product domains. These values provide the quantitative basis for interpreting the domain structure observed in Figure 1 and Figure 2.

The standard deviations provide an estimate of within-domain dispersion for the grades represented in the analyzed portfolio. The homopolymer domains exhibit the expected increase in mean MFI from H-LF to H-HF. However, the relatively broad dispersion of H-HF indicates that this domain covers a wider processability range. Among the random copolymers, R-ME is the most populated domain and therefore provides the most representative description of the central random-copolymer region in this portfolio. R-LE contains only three grades; consequently, its low-ethylene composition and associated property profile should be interpreted only as descriptive characteristics of these specific materials rather than as a statistically established low-ethylene random-copolymer population.

Within the impact-copolymer family, the more populated C-STD and C-HI domains permit a comparatively stronger interpretation of the rubber-phase and impact-related trends. In contrast, C-HMF contains only two grades. Its high mean MFI, therefore, reflects the characteristics of two specialized high-flow impact copolymers in the investigated portfolio but does not define a general MFI range or threshold for this class of polypropylene. Overall, the domain statistics describe the internal organization of the analyzed portfolio and should not be generalized beyond it without additional grades and independent external datasets.

Table 4 confirms that the molecular origin of domain differentiation varies by family. In the homopolymer series, the dominant differentiating parameter is MFI. The increase from H-LF to H-HF reflects a transition from higher-molecular-weight, lower-flow materials to grades designed for greater processability. Since homopolymers do not contain ethylene or rubber-phase descriptors, their differentiation is governed mainly by molecular-weight engineering.

Within the random-copolymer family, the domain means indicate increasing total ethylene content and XS from the lower- to the higher-ethylene regions. This tendency is consistent with ethylene’s role as a comonomer that disrupts isotactic polypropylene crystallinity, increases the amorphous contribution, and modifies optical and mechanical behavior [8,9,28,31]. The trend is most clearly supported by the more populated R-ME domain and its comparison with R-HE. Because R-LE contains only three grades, its position should be regarded as a descriptive low-ethylene reference within this portfolio rather than as a statistically robust endpoint of a universal compositional progression.

Impact copolymers exhibit a distinct molecular signature associated with Rubber EC and Rubber FC, reflecting the importance of the heterophasic elastomeric phase in their portfolio-level differentiation. The comparatively populated C-STD and C-HI domains support the interpretation of a progression toward more toughness-oriented compositions and performance profiles [3,10,11,36]. The two C-HMF grades combine high MFI with measurable rubber-phase descriptors and impact-related performance. However, this observation should be treated as a descriptive feature of these specialized grades and not as evidence of a generally established high-flow impact-copolymer domain.

### 3.2. Chemometric Discrimination of Molecular Architectures

Principal component analysis was performed to assess whether the experimentally measured descriptors support the portfolio’s industrial classification. Figure 2 shows the PCA score plot obtained from the autoscaled structure–property matrix, while Table 5 summarizes the explained variance.

Because Rubber EC and Rubber EC are structural-zero descriptors for homopolymer and random copolymer grades, the PCA score distribution should be interpreted as a portfolio-level latent structure that combines family-defining compositional differences with mechanical, thermal, optical, and processability gradients.

The first two principal components explain 83.8% of the total variance, indicating that most of the portfolio variability is captured in a two-dimensional latent space. This result is significant because the original dataset integrates descriptors with different physical meanings and units, including flow behavior, compositional parameters, phase morphology, mechanical properties, thermal resistance, and optical response.

The high cumulative variance indicates that the selected descriptors capture a substantial fraction of the dominant molecular, compositional, and performance gradients present within the analyzed portfolio. From a chemometric perspective, this supports the use of dimensionality reduction to explore latent structure in the correlated industrial dataset [21,22,28].

The PCA score plot reveals a clear separation among molecular domains. Impact copolymers occupy a distinct region of the multivariate space, reflecting the influence of rubber-phase descriptors and impact-related performance. Homopolymer domains cluster separately, consistent with their absence of ethylene and rubber-phase contributions and their stronger association with crystalline matrix properties. Random copolymers occupy another region, reflecting the effect of ethylene incorporation and XS on crystallinity, optical response, and ductility.

This separation is chemically meaningful, but it should be interpreted as exploratory evidence of latent portfolio structure rather than as independent validation of the predefined domains. PCA does not create or prove the industrial classification; instead, it indicates that the measured descriptor matrix is consistent with a low-dimensional organization reflecting family-level molecular architecture, phase composition, and performance gradients.

**Figure 3:**
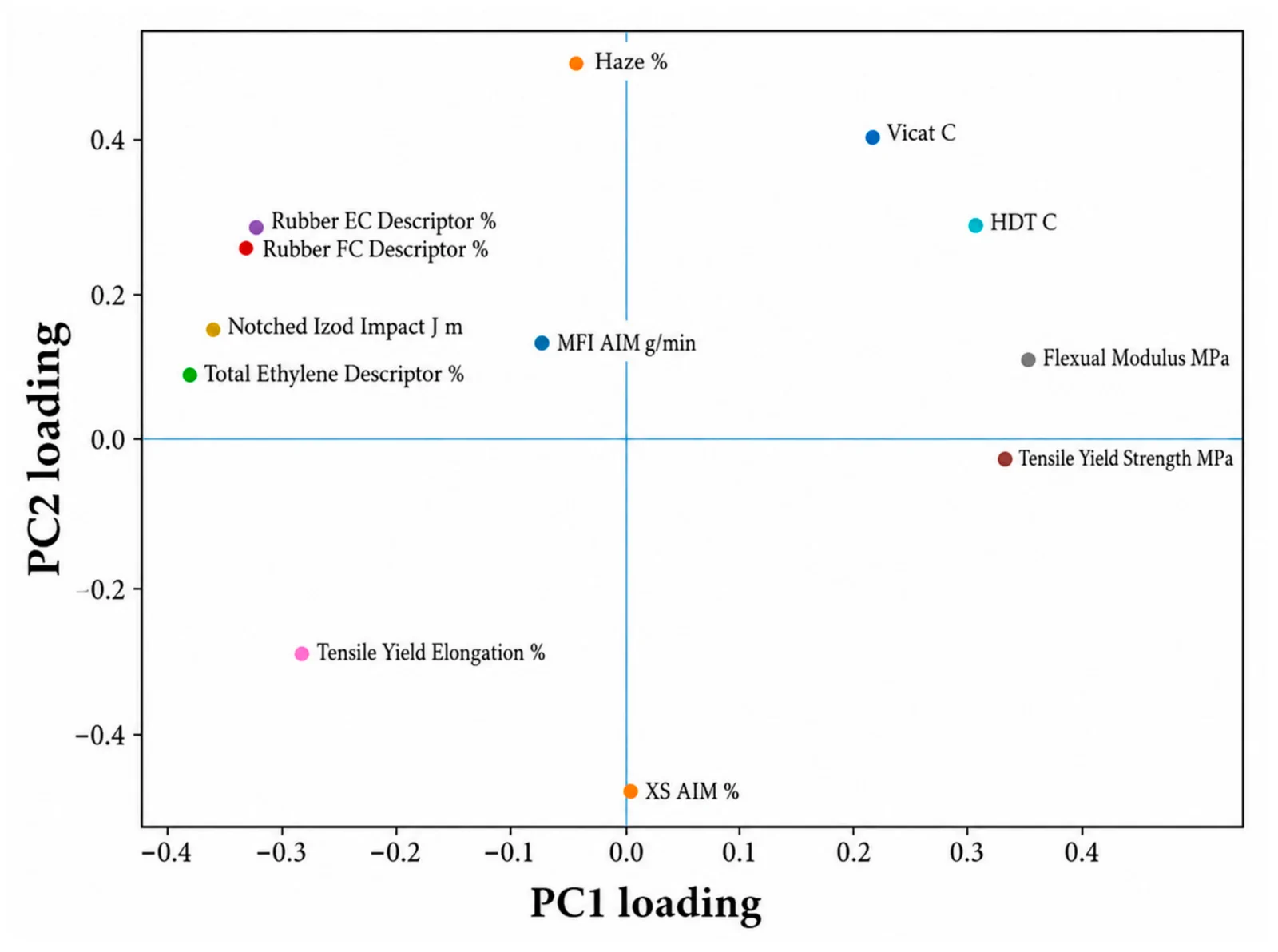
PCA loading plot showing the contribution of molecular/compositional, phase-composition, mechanical, thermal, and optical descriptors to portfolio differentiation. Label size and vector annotations were adjusted to improve readability.

The variables contributing to the PCA separation are shown in Figure 3.

The loading plot was interpreted as an exploratory map of descriptor contributions to the PCA space. Because PCA is unsupervised, the loading structure should be used to interpret descriptor gradients and family-level tendencies, not to infer causal relationships or to validate product domains independently. The loading plot clarifies the molecular origin of the score distribution observed in Figure 2. Descriptors associated with stiffness and thermal resistance are positioned opposite to variables related to ethylene incorporation, rubber-phase morphology, and impact performance. This configuration reflects a fundamental structure–property trade-off in polypropylene design: increasing comonomer or elastomeric-phase content improves toughness and ductility but tends to reduce stiffness and tensile strength [3,10,11,28,29].

Rubber EC, Rubber FC, total ethylene content, and notched Izod impact are located in the same general region of the loading space, indicating that the elastomeric phase composition and content govern toughness-oriented domains. Conversely, tensile strength, flexural modulus, Vicat, and HDT are associated with the more crystalline and mechanically rigid side of the portfolio. These variables characterize the performance space of homopolymer-rich domains, in which load-bearing capacity is governed by molecular weight, crystalline phase integrity, and chain regularity [2,5,6,7].

XS and haze provide an additional layer of interpretation. XS is related to the amorphous fraction and crystallinity reduction, whereas haze reflects the optical consequence of crystal morphology and phase heterogeneity. Their positions indicate that optical behavior cannot be treated as a simple secondary consequence of mechanical performance. Instead, optical response depends on how crystallinity, amorphous content, crystallite size, and phase morphology are modified within each polypropylene family [28,30].

### 3.3. Supervised Classification and Variable Importance

After the unsupervised PCA, PLS-DA was used to quantify the consistency between the experimental descriptor matrix and the three predefined industrial polypropylene families. The analysis was not designed to establish an externally validated classifier for unknown commercial grades. Instead, it evaluated whether molecular, compositional, phase-related, and performance descriptors reproduced the existing separation among homopolymers, random copolymers, and impact copolymers. This distinction is necessary because total ethylene content, Rubber EC, and Rubber FC are directly associated with the molecular definitions of the three families and therefore facilitate supervised discrimination.

**Figure 4:**
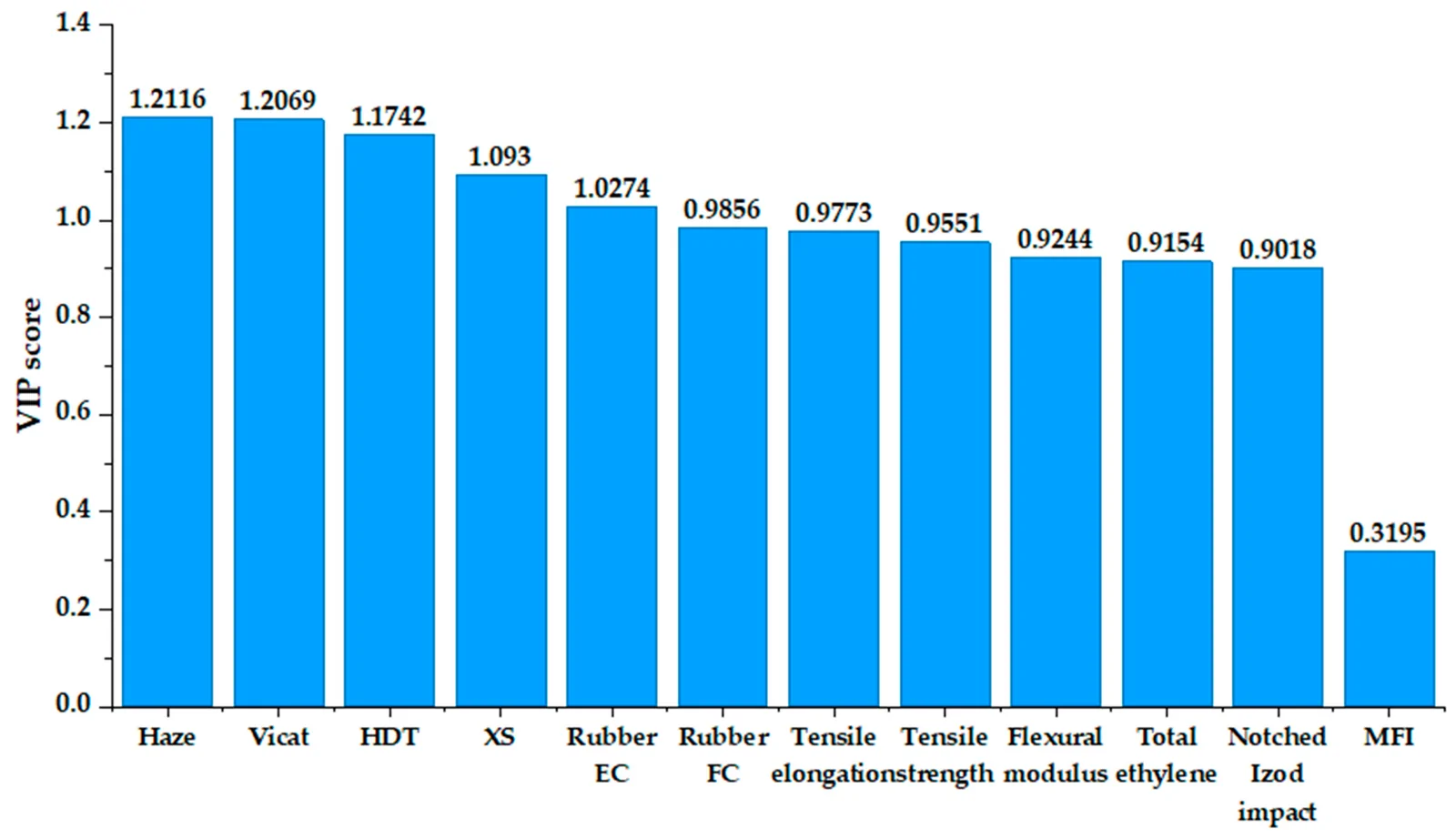
Variable importance in projection (VIP) scores obtained from the PLS-DA model. Variables with VIP scores above 1.0 were considered the main contributors to supervised discrimination. VIP scores were calculated from the fitted full-descriptor PLS-DA model and are reported as model-derived variable-importance-in-the-PLS (VIP) scores. Because no bootstrap-based VIP stability analysis was performed, unsupported error bars were not added to the VIP plot.

The resulting VIP scores are shown in Figure 4.

To evaluate the contribution of the different descriptor groups to internal family discrimination, two PLS-DA models were compared. The molecular/compositional model included only MFI, XS, total ethylene content, Rubber EC, and Rubber FC. In contrast, the full-descriptor model additionally included tensile strength, tensile elongation, HDT, Vicat, and haze. Both models were constructed using two latent variables and evaluated using stratified 5-fold cross-validation. This comparison enabled assessment of whether molecular and phase-composition variables already captured the consistency with the predefined family labels or were further reinforced by downstream mechanical, thermal, and optical performance descriptors.

The corresponding VIP scores are summarized in Table 6.

The VIP scores were interpreted with the understanding that some descriptors, particularly RUBBER EC and Rubber FC, are structurally absent outside the impact-copolymer family. Therefore, their contribution reflects both the molecular relevance of the elastomeric phase and its role in discriminating heterophasic materials from non-heterophasic polypropylene grades.

The VIP analysis indicates that the combined contribution of optical, thermal, amorphous-phase, and elastomer-composition descriptors drives the supervised discrimination of the portfolio. Haze, Vicat, HDT, XS, and Rubber EC exceed the conventional VIP threshold of 1.0, identifying them as the most influential variables in the PLS-DA model [24,32,33,34,35]. RUBBER EC is slightly below this threshold but remains close to unity, indicating a relevant secondary contribution to supervised discrimination.

This result is important because it refines the interpretation of the molecular drivers. Although RUBBER FC and RUBBER EC are chemically central to the design of impact copolymers, the overall classification of the complete portfolio is not determined exclusively by rubber-phase parameters. Instead, the PLS-DA model integrates optical scattering, thermal resistance, amorphous content, and elastomer composition into a broader discriminatory structure. The high VIP score for haze suggests that the optical response is sensitive not only to random copolymer crystallinity but also to phase morphology across the entire portfolio. Similarly, the strong contribution of Vicat and HDT indicates that thermal resistance remains a key discriminator between crystalline homopolymer-rich regions and compositionally modified domains.

The low VIP score of MFI indicates that melt flow is not the main variable separating the three molecular families, even though it is essential for differentiating processing grades within a given family. This behavior is chemically coherent because MFI primarily reflects differences in molecular weight and processability, whereas family-level discrimination depends more strongly on composition, morphology, optical response, and thermal behavior.

**Figure 5:**
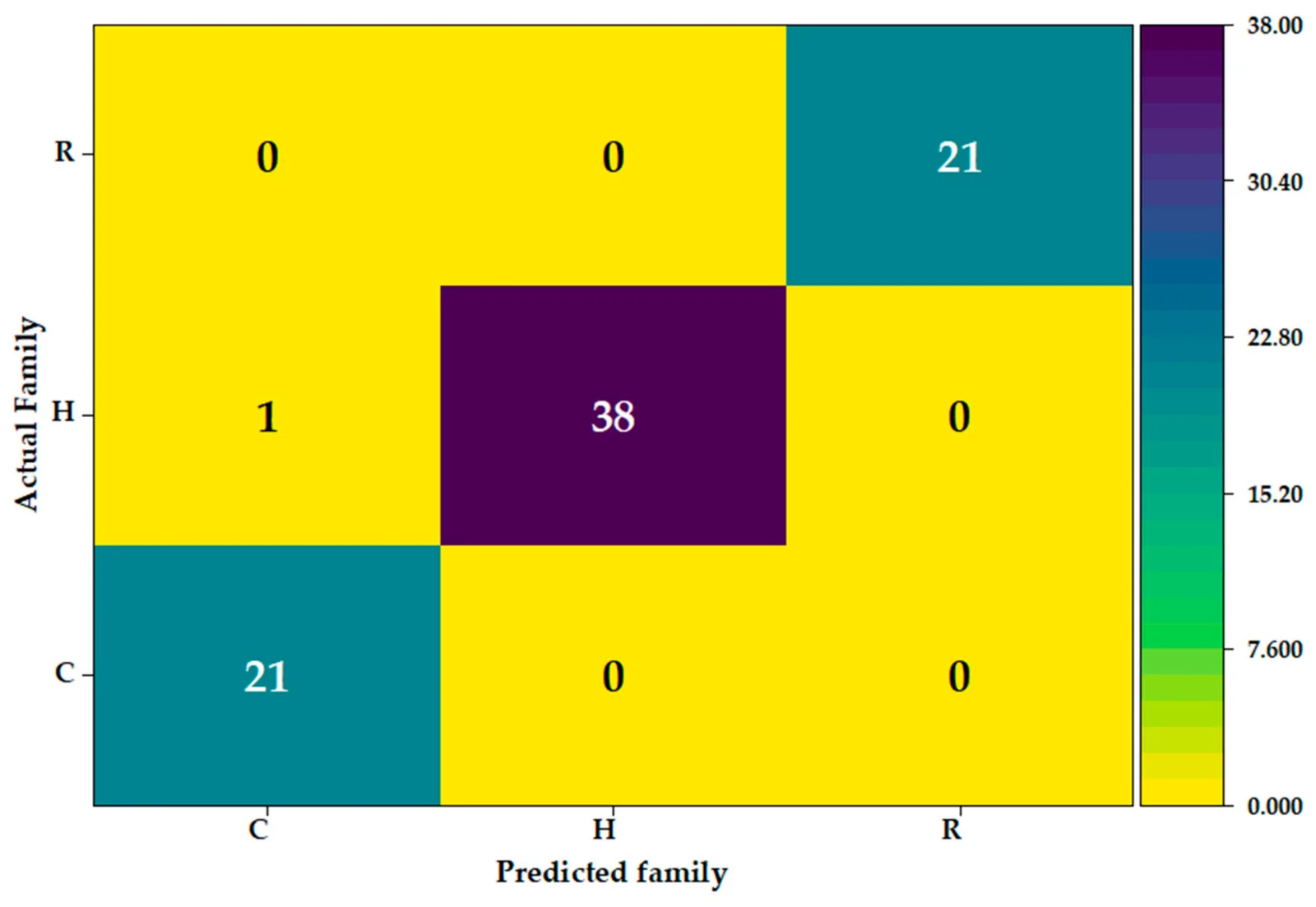
Confusion matrix obtained from the full-descriptor PLS-DA model under stratified 5-fold cross-validation. Rows represent the predefined industrial polypropylene families, and columns represent the corresponding model assignments. C, H, and R denote impact copolymer, homopolymer, and random copolymer, respectively. The matrix evaluates internal agreement with the existing family classification and does not represent external predictive validation.

The classification performance of the full-descriptor PLS-DA model is shown in Figure 5 and summarized in Table 7.

The molecular/compositional PLS-DA model achieved 93.8% calibration and cross-validated accuracy, with 76 of the 81 grades consistently assigned to their predefined industrial family. This result shows that molecular and phase-composition descriptors reproduce most of the existing family organization. However, this performance is partly expected because total ethylene content, Rubber EC, and Rubber FC are structurally related to the distinctions among homopolymer, random copolymer, and impact copolymer materials.

The full-descriptor PLS-DA model reached 98.8% calibration and cross-validated accuracy, assigning 80 of the 81 grades consistently with their industrial family labels. Cross-validated precision, recall, and F1-score were also 98.8%. The increase relative to the molecular/compositional model indicates that mechanical, thermal, and optical properties provide additional information that is consistent with the molecular architecture and morphology of the known families. Nevertheless, these metrics quantify internal agreement with the existing classification and should not be interpreted as externally validated predictions of unknown polypropylene grades.

The permutation test yielded *p* = 0.002 for both models based on 500 random permutations of the family labels, indicating that the observed discrimination was unlikely to arise from random class assignment alone. This test supports the statistical significance of the internal supervised separation but does not establish external predictive validity. The result must therefore be considered together with the structural relationship between several predictors and the predefined family labels. In particular, total ethylene content, Rubber EC, and Rubber FC directly distinguish materials with no comonomer, random ethylene incorporation, or a heterophasic elastomeric phase.

The single inconsistent assignment corresponds to a grade whose descriptor profile approaches the neighboring family region in the latent-variable space. This behavior may reflect an intermediate performance profile or partial overlap between the measured properties of adjacent industrial families. Because the grade’s identity was evaluated within the same producer-specific portfolio, this case should be interpreted descriptively rather than as evidence of general classification behavior for boundary grades in other commercial datasets.

### 3.4. Structure–Property Correlations Across the Portfolio

**Figure 6:**
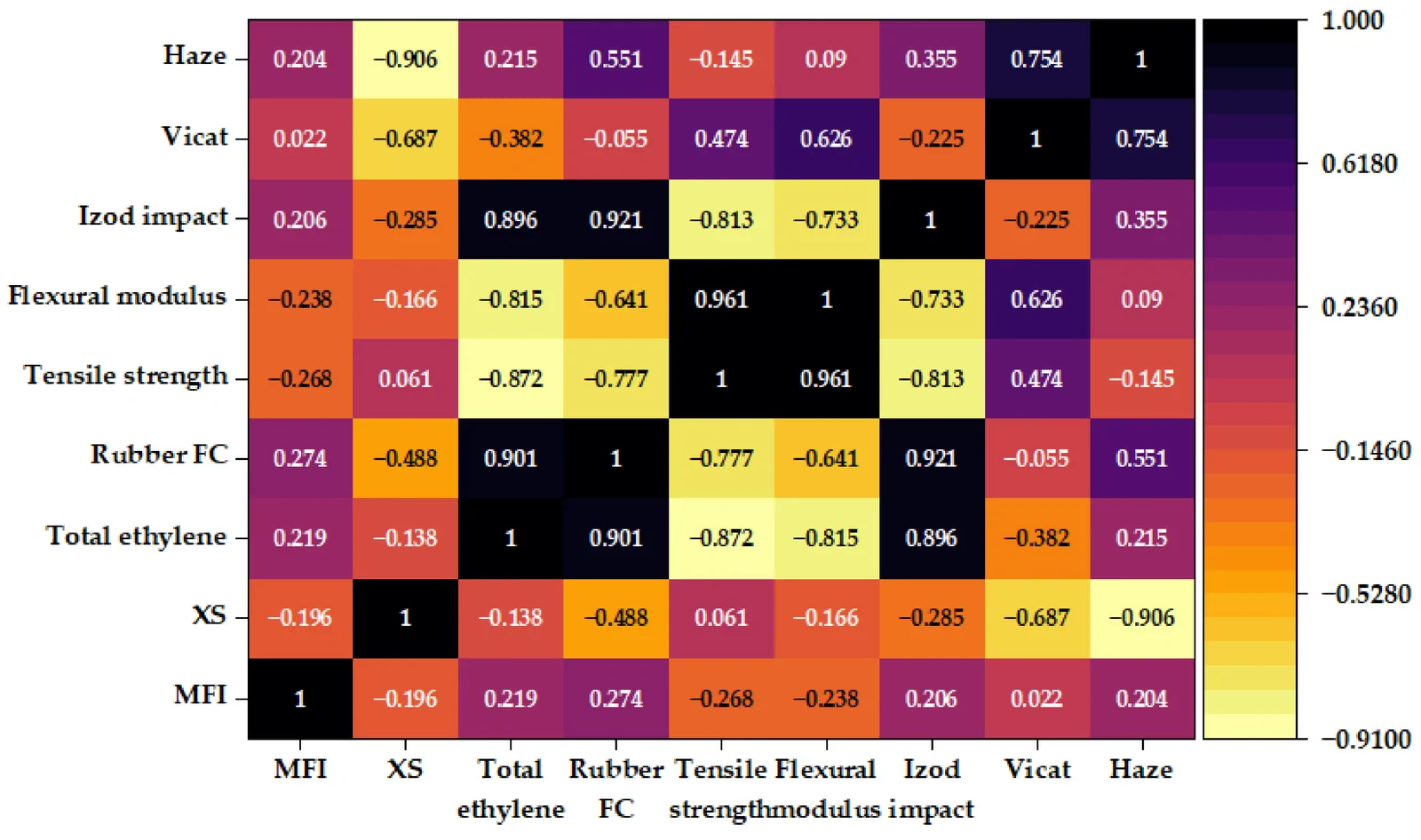
Pearson correlation heatmap showing pairwise relationships among molecular descriptors and performance variables in the industrial polypropylene portfolio. Positive correlations indicate variables that increase together, whereas negative correlations indicate inverse structure–property trends.

Pearson correlation analysis was used to quantify pairwise relationships among molecular descriptors and performance variables. The resulting correlation heatmap is shown in Figure 6.

The Pearson correlation heatmap should be interpreted as a portfolio-level association map. Because the dataset combines homopolymers, random copolymers, and impact copolymers, some correlations may be influenced by between-family differences, including structural-zero descriptors, rather than representing universal within-family structure–property relationships. Within this scope, the correlation heatmap indicates well-defined molecular trade-offs across the portfolio. Rubber FC shows a strong positive association with notched Izod impact at the portfolio level. Consistent with the expected role of the rubber phase in increasing impact resistance in heterophasic polypropylene. However, because Rubber EC is structurally absent outside the impact-copolymer family, this correlation should be interpreted as reflecting both the family-level separation of impact copolymers and the contribution of Rubber EC to toughness within the heterophasic design space [3,11,12,38].

However, the same variables that enhance impact performance are negatively associated with tensile strength and flexural modulus. This inverse relationship is consistent with the classical stiffness–toughness compromise observed in polypropylene modification. In the present dataset, this trend should be interpreted at the portfolio level, as it encompasses differences among homopolymers, random copolymers, and impact copolymers, rather than isolating a single within-family mechanism [3,10,35,36].

Tensile strength and flexural modulus show a strong positive relationship, indicating that rigidity and tensile resistance evolve together in more crystalline or less compositionally modified regions of the portfolio. This behavior is consistent with the homopolymer domains, in which mechanical performance is governed primarily by molecular weight, isotactic sequence regularity, and crystalline phase integrity [2,5,6,7].

The XS–haze relationship requires a family-dependent interpretation. Optical scattering depends not only on amorphous content but also on crystallite size, crystal perfection, refractive-index mismatch, and phase heterogeneity. In random copolymers, ethylene-induced disruption of crystallinity can reduce light scattering and improve transparency. In impact copolymers, however, dispersed rubber domains can increase opacity through morphological heterogeneity. Therefore, the relationship between XS and haze should be interpreted within each molecular family rather than as a universal single-variable rule [28,29].

### 3.5. Domain-Level Structure–Property Profiles

To integrate the molecular, compositional, and performance characteristics of the portfolio, the nine predefined product domains were interpreted using standardized domain-level profiles and are summarized in Table 8. The complete standardized heatmap is provided as Figure S1 in the Supplementary Materials. Application spaces are included only as contextual descriptions of the established industrial positioning of each domain. Because R-LE and C-HMF contain only three and two grades, respectively, their profiles should be treated as descriptive summaries of specialized portfolio niches rather than as statistically robust or broadly transferable domain estimates.

Table 8 shows that each domain is associated with a distinct molecular or compositional design strategy. Homopolymer domains are governed mainly by molecular-weight-related flow control. In this family, the transition from H-LF to H-HF reflects a shift from stiffness- and melt-strength-oriented grades toward higher processability and productivity. Because these materials do not contain comonomer or rubber-phase descriptors, their differentiation is controlled primarily by MFI and the associated molecular-weight-related processing window.

Random copolymer organization is primarily associated with ethylene incorporation and the resulting modification of the crystalline morphology. Across the analyzed grades, increases in total ethylene content and XS are consistent with progressive disruption of isotactic polypropylene crystallinity and corresponding changes in optical and mechanical behavior [8,9,35,36,37]. R-ME, which contains 13 grades, provides the most representative description of the balanced random-copolymer region in this portfolio, whereas R-HE represents a more compositionally modified region. The R-LE profile is based on only three grades and is therefore retained as a descriptive low-ethylene niche rather than as a statistically broad domain.

Impact-copolymer domains exhibit the strongest rubber-phase and impact-response signatures, consistent with the established role of heterophasic morphology in energy dissipation and toughness development [3,11,12,38]. The C-STD and C-HI domains, represented by 13 and 7 grades, respectively, support the interpretation of distinct standard- and higher-impact regions within the analyzed portfolio. C-HMF contains only two grades; therefore, its combination of high MFI and impact-copolymer characteristics is reported as an observed profile of a specialized portfolio niche rather than as a statistically established domain pattern.

**Figure 7:**
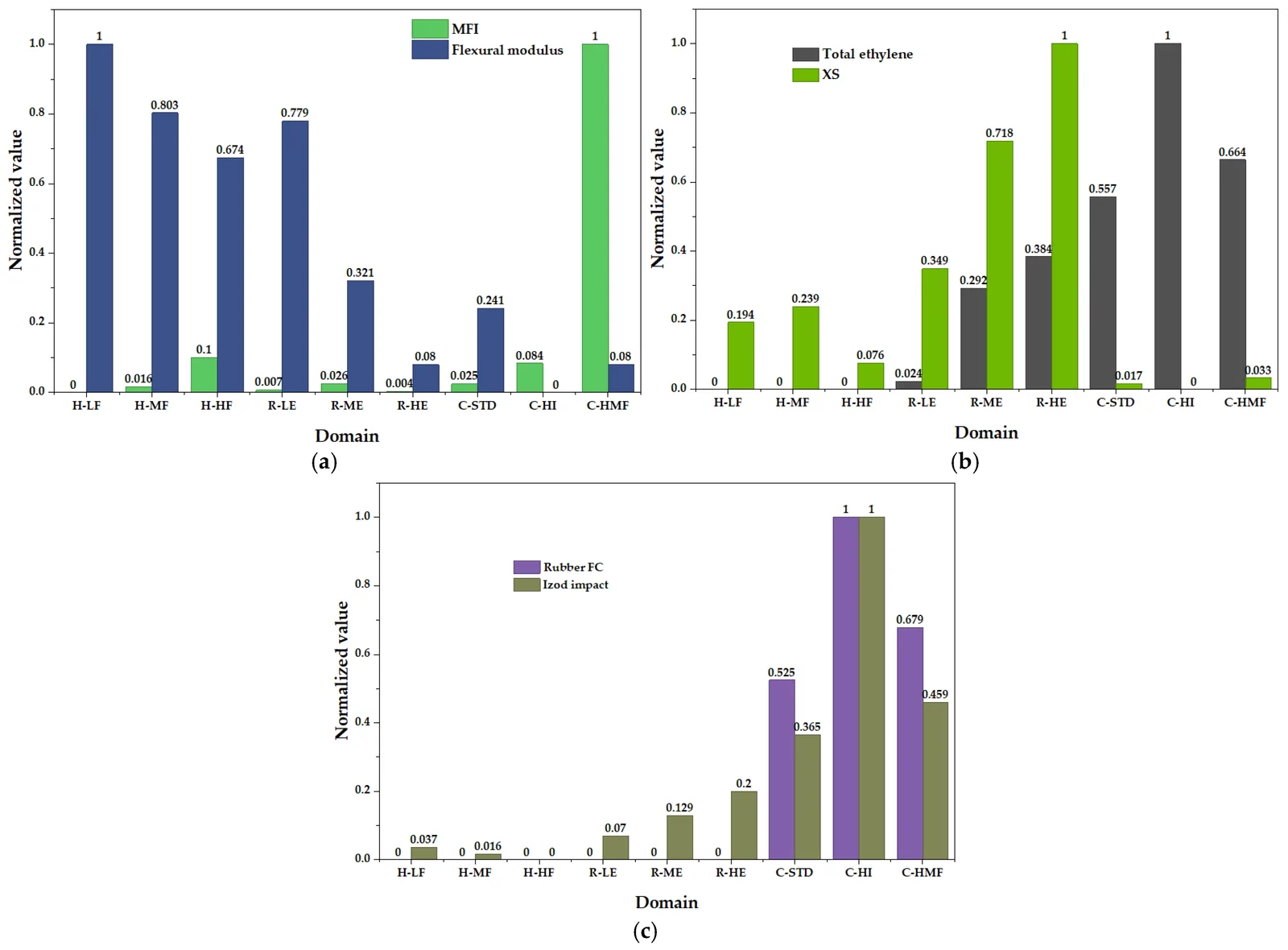
Normalized descriptor profiles across the predefined polypropylene product domains, represented as grouped bar plots. Values were min–max normalized to compare descriptors with different units on a common 0–1 scale. Domains are ordered according to family sequence: H-LF, H-MF, H-HF, R-LE, R-ME, R-HE, C-STD, C-HI, and C-HMF. Panel (**a**) compares MFI and flexural modulus as indicators of the flow–stiffness profile. Panel (**b**) compares total ethylene content and XS as compositional and amorphous-phase descriptors. Panel (**c**) compares Rubber FC and notched Izod impact as indicators of the rubber-phase–impact profile. MFI, melt flow index; XS, xylene-soluble fraction; Rubber FC, rubber-phase fraction.

To visualize the main descriptor trajectories more clearly, Figure 7 represents selected normalized molecular and performance profiles across the same domain sequence. This representation complements Table 8 by showing how flow behavior, stiffness, ethylene incorporation, amorphous contribution, Rubber EC, and impact response evolve across the portfolio. The grouped-bar format was selected to improve readability relative to the previous normalized profile representation and to allow direct comparison among domains ordered by polypropylene family.

Figure 7 summarizes the normalized descriptor trajectories observed across the predefined domains. The homopolymer profiles show a progression from lower-flow, stiffness-oriented grades toward higher-flow grades. In contrast, the random-copolymer profiles reflect differences in total ethylene content and XS consistent with ethylene-induced modification of crystalline organization [9,10,29,30,31]. The impact-copolymer profiles show the strongest contributions from rubber-phase and impact-related descriptors, in agreement with the role of heterophasic morphology in toughness development [3,10,11,29]. However, the normalized values for R-LE and C-HMF are based on only three and two grades, respectively. Their apparent positions and extrema should therefore be interpreted descriptively and should not be used to define generalized domain trajectories or numerical thresholds. In particular, the maximum normalized MFI of C-HMF reflects the two grades included in this portfolio rather than a universal characteristic of high-flow impact polypropylene.

The industrial-use contexts considered in this study include raffia, fibers, films, thermoforming, injection molding, packaging, household products, appliances, and automotive applications. These categories correspond to the established commercial positioning of the analyzed grades and were not inferred from the PCA or PLS-DA results. Accordingly, the study does not propose an independent chemometric model for predicting the final application of an unknown polypropylene grade. Instead, the application information provides industrial context for interpreting the molecular and performance characteristics of the predefined domains [32].

Polypropylene is also used in membrane-related applications because of its chemical resistance, hydrophobicity, processability, and stability under several separation environments. However, membrane-specific grades were not represented as a separate domain in the present portfolio. Consequently, no conclusion regarding membrane-grade classification or performance can be derived from the current dataset.

The combined domain-level and chemometric analyses indicate that the investigated portfolio follows a chemically interpretable organization. Homopolymer grades are differentiated mainly by molecular-weight-related flow behavior, random copolymers by ethylene-induced modification of crystalline organization, and impact copolymers by heterophasic rubber-phase characteristics. PCA revealed an interpretable low-dimensional descriptor structure, whereas PLS-DA quantified the consistency between the experimental matrix and the known industrial family labels. VIP and correlation analyses identified the variables contributing to this family-level organization and the principal portfolio-scale property trade-offs. These results support the use of the proposed workflow for internal portfolio rationalization and grade comparison. They do not, however, establish universal domain boundaries, independent application prediction, or cross-supplier classification capability.

### 3.6. External Consistency and Transferability of the Domain Framework

The molecular trends identified in this work are consistent with established polypropylene design principles. The three main strategies observed within the analyzed portfolio—molecular-weight-related flow control in homopolymers, ethylene incorporation in random copolymers, and rubber-phase engineering in impact copolymers—agree with well-established polypropylene structure–property relationships [3,9,10,11,12,29,30,31]. These mechanisms provide a scientifically coherent basis for interpreting the molecular, compositional, and performance gradients detected by the chemometric analyses. However, the resulting domain boundaries should not be regarded as universal numerical limits for all commercial polypropylene portfolios.

For homopolymer grades, the transition from low- to high-flow domains is governed primarily by MFI, which acts as an indirect descriptor of molecular-weight-related processability. This strategy enables adjustment of processing behavior while preserving the stiffness and thermal resistance of a semicrystalline polypropylene matrix. In random copolymers, ethylene incorporation represents the principal compositional variable controlling the disruption of chain regularity and the resulting balance among transparency, ductility, and stiffness. In impact copolymers, Rubber EC and Rubber FC describe, respectively, the ethylene content and relative fraction of the elastomeric phase, which strongly influence toughness and impact-energy dissipation in heterophasic polypropylene materials [3,10,11,29].

Nevertheless, the present analysis was conducted using a single industrial polypropylene portfolio. Consequently, the reported domain populations, descriptor ranges, and numerical boundaries should be considered specific to the analyzed producer and its product-design strategy. Although the dataset includes 21 random copolymer grades distributed across low-, medium-, and high-ethylene domains, these materials share a common industrial origin. They may reflect producer-specific catalyst technology, reactor configuration, formulation practices, and commercial specifications. The current results therefore demonstrate internal consistency within the investigated portfolio but do not establish cross-supplier validity.

The transferable contribution of the study lies in the methodological workflow rather than in the exact numerical classification of the nine domains. The integration of molecular and compositional descriptors, performance variables, autoscaling, PCA, PLS-DA, VIP analysis, Pearson correlation analysis, and domain-level profiling can be applied to independent polypropylene datasets. However, the portability of the framework must be evaluated using external portfolios containing grades manufactured by different producers and under different catalyst systems, reactor technologies, molecular-weight distributions, and formulation strategies. Application categories should therefore be interpreted as contextual industrial information associated with the predefined domains rather than as outcomes independently predicted by the chemometric models.

Extrapolation of the present results to final-use polypropylene products must also account for formulation effects. Commercial polypropylene materials commonly contain stabilizers, antioxidants, nucleating agents, slip agents, antistatic agents, pigments, mineral fillers, impact modifiers, and processing aids. These components can modify crystallization behavior, stiffness, toughness, optical response, thermal stability, degradation resistance, and rheological performance. Accordingly, the reported profiles should be interpreted as portfolio-level descriptions of the analyzed commercial grades rather than as complete predictors of formulated-product behavior under all processing and service conditions.

Future studies should evaluate the framework using independent polypropylene portfolios from different producers, with particular attention to random copolymers manufactured using different catalyst technologies and comonomer-control strategies. Incorporating additive-package information, molecular-weight-distribution measurements, oscillatory and capillary rheology, real-time process variables, and external validation datasets would permit a more rigorous assessment of the numerical domain boundaries and model transferability. Such validation would strengthen the workflow’s potential for portfolio rationalization, grade comparison, product development, and data-driven polymer design.

## 4. Conclusions

Chemometric analysis of 81 industrial polypropylene grades revealed a low-dimensional organization consistent with the predefined homopolymer, random copolymer, and impact copolymer families. The first two principal components explained 83.8% of the total variance. Homopolymer differentiation was associated mainly with molecular-weight-related flow behavior, random-copolymer organization with total ethylene content and XS, and impact-copolymer differentiation with Rubber EC, Rubber FC, and impact-related performance. These results support a portfolio-specific structure–property interpretation rather than a universal classification of commercial polypropylene grades.

The molecular/compositional PLS-DA model achieved 93.8% cross-validated accuracy, whereas the full-descriptor model reached 98.8% using two latent variables, assigning 80 of the 81 grades consistently with their predefined family labels. These values reflect internal agreement with the existing industrial classification, not independently validated predictive capability, because several descriptors are structurally related to the family definitions. Application categories were predefined industrial metadata and were not derived from the chemometric models. Moreover, R-LE and C-HMF, represented by three and two grades, respectively, should be interpreted only as descriptive portfolio niches. Because all grades originated from a single producer, the numerical domain boundaries and model transferability require validation using independent polypropylene portfolios.

## 5. Limitations and Future Perspectives

The principal limitation of this study is that all 81 grades were obtained from a single industrial polypropylene portfolio. Although the descriptor trends are consistent with established molecular design principles, the reported domain boundaries, grade distributions, and classification results may reflect producer-specific catalyst technologies, reactor configurations, formulation strategies, and commercial specifications. Therefore, the present results establish internal portfolio consistency but do not demonstrate cross-supplier transferability.

A second limitation concerns formulation complexity. The present framework was built from the molecular/compositional and performance descriptors available for the analyzed grades. However, it does not explicitly resolve the effect of stabilizers, antioxidants, nucleating agents, pigments, fillers, processing aids, or other additive packages commonly used in commercial polypropylene products. These additives may shift crystallization behavior, stiffness, toughness, optical response, thermal stability, degradation resistance, and processability. Therefore, the proposed maps should be interpreted as portfolio-level structure–property–application descriptors of the analyzed industrial grades, not as complete predictors of all formulated polypropylene systems under real service conditions. Commercial polypropylene materials are frequently formulated with stabilizers, antioxidants, nucleating agents, slip agents, antistatic agents, pigments, mineral fillers, flame retardants, impact modifiers, or other additives. These components can modify crystallization behavior, stiffness, toughness, optical response, thermal stability, degradation resistance, flammability, and processing performance [40].

The present analysis used MFI as an industrial descriptor of molecular-weight-related processability. Although this parameter is highly relevant for routine grade differentiation, it does not capture the full viscoelastic behavior of polypropylene melts, including shear-thinning response, melt elasticity, elongational viscosity, relaxation dynamics, and molecular weight distribution effects [8]. Therefore, the rheological interpretation of the portfolio should be understood as processability-oriented rather than as a full melt-rheology analysis.

The PLS-DA models were validated internally using stratified 5-fold cross-validation and permutation testing. However, no independent external test set was available because the study was restricted to a single industrial portfolio. Therefore, the reported classification performance should be interpreted as internal evidence of consistency between the descriptor matrix and the known industrial family structure, rather than as externally validated predictive performance for unknown polypropylene grades or other commercial portfolios.

Future studies should evaluate the workflow using independent polypropylene portfolios from different producers, with particular attention to random copolymer grades manufactured using different catalyst systems and comonomer-control strategies. External test sets, molecular-weight-distribution data, oscillatory and capillary rheology, additive-package information, and process-history variables would enable a more rigorous assessment of transferability. Such validation is required before the numerical domain boundaries or the PLS-DA performance reported here can be used to predict the classification of unknown commercial polypropylene grades.

## Figures and Tables

**Table 1:** Composition of the industrial polypropylene portfolio investigated in this study.

Family	Number of Grades	Percentage (%)	General Molecular Characteristics
Homopolymer	38	46.9	High crystallinity and absence of comonomer incorporation
Random copolymer	21	25.9	Ethylene-modified polypropylene with reduced crystallinity
Impact copolymer	22	27.2	Heterophasic polypropylene containing an elastomeric rubber phase
Total	81	100.0	Industrial polypropylene portfolio

**Table 2:** Molecular, compositional, phase-composition, mechanical, thermal, and optical descriptors were employed in the chemometric analysis of the industrial polypropylene portfolio.

Descriptor	Category	Unit	Molecular, Compositional, or Technological Meaning
MFI	Molecular/processability proxy	g/10 min	Indirect indicator of molecular-weight-related melt flow and processability
XS	Compositional/morphological	wt.%	Xylene-soluble fraction; proxy for amorphous contribution and crystallinity disruption
Total ethylene content	Compositional	wt.%	Ethylene incorporation in random and impact copolymer structures
Rubber EC	Phase-composition	wt.%	Ethylene content of the elastomeric rubber phase in impact copolymers
Rubber FC	Phase-composition	wt.%	Relative fraction of the elastomeric rubber phase in impact copolymers
Tensile strength	Mechanical performance	MPa	Resistance under tensile loading
Tensile elongation	Mechanical performance	%	Ductility under tensile deformation
Flexural modulus	Mechanical performance	MPa	Stiffness under flexural loading
Notched Izod impact	Mechanical performance	J/m	Resistance to impact fracture
Vicat	Thermal performance	°C	Softening-temperature response
HDT	Thermal performance	°C	Heat deflection temperature under load
Haze	Optical performance	%	Light-scattering response and optical clarity

**Table 3:** Portfolio-specific operational criteria are used for assigning polypropylene grades to product domains. Domain assignment was based on industrial product specifications, commercial positioning, molecular architecture, flow behavior, comonomer incorporation, elastomeric-phase descriptors, performance profile, and intended application space.

Domain	Full Domain Name	Family	Primary Assignment Basis	Secondary Assignment Criteria	Main Application Logic	Interpretation Note
H-LF	Low-flow homopolymer	Homopolymer	Low MFI within the homopolymer family	Absence of ethylene and rubber-phase descriptors; high stiffness/strength profile	Raffia, fibers, extrusion-oriented applications	Assigned mainly by molecular-weight-related flow behavior, interpreted as a higher-molecular-weight homopolymer region
H-MF	Medium-flow homopolymer	Homopolymer	Intermediate MFI within the homopolymer family	Absence of ethylene and rubber-phase descriptors; balanced stiffness/processability	General injection molding and balanced homopolymer applications	Represents the central homopolymer platform of the portfolio
H-HF	High-flow homopolymer	Homopolymer	High MFI within the homopolymer family	Absence of ethylene and rubber-phase descriptors; processability-oriented profile	High-productivity or thin-wall injection molding	Assigned by high-flow requirement rather than compositional modification
R-LE	Low-ethylene random copolymer	Random copolymer	Low ethylene incorporation within the random copolymer family	Moderate XS; random copolymer architecture; stiffness-retaining profile	Packaging, film/sheet applications requiring moderate clarity	Small specialized domain; should not be interpreted as a statistically broad cluster
R-ME	Medium-ethylene random copolymer	Random copolymer	Intermediate ethylene incorporation	Increased XS relative to homopolymers; transparency–toughness balance	Food containers, rigid packaging, thermoforming	Represents the main balanced random copolymer domain
R-HE	High-ethylene random copolymer	Random copolymer	High ethylene incorporation within the random copolymer family	Higher XS; lower stiffness; optical/ductility-oriented profile	High-clarity or ductility-demanding thermoformed/packaging applications	Interpreted as the most compositionally modified random copolymer domain
C-STD	Standard-impact copolymer	Impact copolymer	Presence of a heterophasic rubber phase with moderate Rubber FC	Rubber EC within the impact-copolymer compositional range; standard Izod–stiffness balance	Appliances, industrial products, and standard impact applications	Represents the standard heterophasic platform of the portfolio
C-HI	High-impact copolymer	Impact copolymer	High Rubber FC and a high-impact-resistance profile	Rubber EC consistent with a toughness-oriented elastomeric phase; reduced stiffness relative to homopolymer domains	Automotive and high-toughness applications	Assigned primarily by toughness-oriented heterophasic morphology
C-HMF	High-flow impact copolymer	Impact copolymer	High MFI combined with heterophasic rubber-phase descriptors	Rubber EC and Rubber FC within the impact-copolymer design space, with retained impact-related performance	Thin-wall impact molding, complex injection-molded parts, automotive interiors	Specialized niche domain; high MFI should not be generalized beyond the analyzed portfolio

**Table 4:** Molecular and phase-composition characteristics of the predefined product domains within the industrial polypropylene portfolio. Values are reported as mean ± standard deviation. Zero values for total ethylene content in homopolymers and for Rubber EC and Rubber FC in both homopolymers and random copolymers indicate the structural absence of the corresponding compositional feature. R-LE (*n* = 3) and C-HMF (*n* = 2) are included as descriptive specialized portfolio niches; their mean values and standard deviations should not be interpreted as statistically robust population estimates or universal domain thresholds.

Domain	Family	*n*	MFI (g/10 min)	XS (%)	Total Ethylene Content (%)	Rubber EC (%)	Rubber FC (%)
H-LF	Homopolymer	10	2.06 ± 0.72	2.98 ± 0.37	0.00 ± 0.00	0.00 ± 0.00	0.00 ± 0.00
H-MF	Homopolymer	18	4.56 ± 2.22	3.36 ± 1.18	0.00 ± 0.00	0.00 ± 0.00	0.00 ± 0.00
H-HF	Homopolymer	10	17.40 ± 7.60	1.97 ± 0.84	0.00 ± 0.00	0.00 ± 0.00	0.00 ± 0.00
R-LE	Random copolymer	3	3.15 ± 0.35	4.30 ± 0.00	0.33 ± 0.58	0.00 ± 0.00	0.00 ± 0.00
R-ME	Random copolymer	13	6.11 ± 4.85	7.45 ± 0.96	4.11 ± 0.45	0.00 ± 0.00	0.00 ± 0.00
R-HE	Random copolymer	5	2.68 ± 0.99	9.86 ± 0.35	5.40 ± 0.27	0.00 ± 0.00	0.00 ± 0.00
C-STD	Impact copolymer	13	5.88 ± 5.61	1.46 ± 0.53	7.83 ± 4.15	44.92 ± 20.16	16.04 ± 6.49
C-HI	Impact copolymer	7	14.97 ± 12.36	1.32 ± 0.12	14.06 ± 0.77	46.43 ± 4.47	30.57 ± 3.72
C-HMF	Impact copolymer	2	155.00 ± 56.57	1.60 ± 0.00	9.34 ± 0.16	45.00 ± 0.00	20.75 ± 0.35

**Table 5:** Explained variance obtained from principal component analysis.

Principal Component	Explained Variance (%)	Cumulative Variance (%)
PC1	51.6	51.6
PC2	32.2	83.8

**Table 6:** VIP scores obtained from the PLS-DA model.

Variable	VIP Score
Haze	1.2116
Vicat	1.2069
HDT	1.1742
XS	1.0930
Rubber EC	1.0274
Rubber FC	0.9856
Tensile elongation	0.9773
Tensile strength	0.9551
Flexural modulus	0.9244
Total ethylene content	0.9154
Notched Izod impact	0.9018
MFI	0.3195

**Table 7:** Internal calibration and cross-validation performance of the PLS-DA models used to evaluate consistency with the predefined polypropylene family classification. The molecular/compositional model included MFI, XS, total ethylene content, Rubber EC, and Rubber FC. The full-descriptor model included these variables together with tensile strength, tensile elongation, HDT, Vicat softening temperature, and haze. The reported metrics correspond to the analyzed industrial portfolio and should not be interpreted as externally validated predictive performance.

Metric	Molecular/Compositional PLS-DA	Full-Descriptor PLS-DA
X variables	MFI, XS, total ethylene content, Rubber EC, Rubber FC	MFI, XS, total ethylene content, Rubber EC, Rubber FC, tensile strength, tensile elongation, HDT, Vicat, haze
Missing-value handling	Domain-median imputation for 3 missing molecular-descriptor entries	Domain-median imputation for 3 missing molecular-descriptor entries
Validation strategy	Stratified 5-fold cross-validation	Stratified 5-fold cross-validation
Latent variables	2	2
Calibration accuracy (%)	93.8	98.8
Cross-validated accuracy (%)	93.8	98.8
Cross-validated precision (%)	94.5	98.8
Cross-validated recall (%)	93.8	98.8
Cross-validated F1-score (%)	93.8	98.8
Correctly classified grades, calibration	76/81	80/81
Correctly classified grades, cross-validation	76/81	80/81
Permutation test	500 random class-label permutations	500 random class-label permutations
Permutation test *p*-value	0.002	0.002

**Table 8:** Molecular design characteristics, expected morphology, performance profile, and predefined industrial-use context of the polypropylene product domains.

Domain	Dominant Molecular/ Compositional Feature	Expected Morphology	Expected Performance Profile	Predefined Industrial-Use Context
H-LF	High molecular weight/low MFI	Highly crystalline PP matrix	High stiffness, strength, and melt strength	Raffia, fibers, extrusion-oriented applications
H-MF	Intermediate molecular weight/medium MFI	Crystalline PP matrix	Balanced stiffness, strength, and processability	General injection molding, household products
H-HF	Lower molecular weight/high MFI	Crystalline PP matrix with high flowability	High processability and productivity, with lower melt strength	Thin-wall or high-productivity injection molding
R-LE	Low ethylene incorporation	Semi-crystalline random copolymer	Moderate clarity and stiffness-retaining behavior	Packaging, films, sheets
R-ME	Medium ethylene incorporation	Reduced-crystallinity random copolymer	Transparency–toughness balance with moderate stiffness	Food containers, rigid packaging, thermoforming
R-HE	High ethylene incorporation	Highly modified crystalline morphology	High transparency and ductility, reduced stiffness	High-clarity packaging and ductility-oriented thermoforming
C-STD	Moderate Rubber FC and heterophasic morphology	PP matrix with dispersed elastomeric phase	Standard impact resistance with acceptable stiffness	Appliances, industrial products, impact-modified packaging
C-HI	High Rubber FC and toughness-oriented heterophasic design	Rubber-rich heterophasic morphology	High toughness and impact-energy dissipation, lower stiffness	Automotive and high-impact applications
C-HMF	High MFI combined with impact-copolymer morphology	High-flow heterophasic morphology	Impact resistance combined with high mold-filling ability	Thin-wall impact molding, complex injection-molded parts, automotive interiors

Note: R-LE and C-HMF contain three and two grades, respectively. Their molecular, morphological, performance, and industrial-use descriptions summarize the observed profiles of these specialized grades and should not be interpreted as statistically robust domain characteristics or transferable classification rules.

## Data Availability

The original contributions presented in this study are included in the article/Supplementary Materials. Further inquiries can be directed to the corresponding author.

## Supplementary Materials

The following supporting information can be downloaded at: https://www.mdpi.com/article/10.3390/polym18162009/s1, Figure S1: Domain-level structure–property heatmap of the industrial polypropylene portfolio. Values correspond to standardized domain means calculated for molecular/compositional, phase-composition, mechanical, thermal, and optical descriptors. Positive values indicate domain means above the portfolio average, whereas negative values indicate domain means below the portfolio average. Domains with small sample sizes, particularly R-LE and C-HMF, should be interpreted descriptively as specialized portfolio niches rather than statistically broad clusters; Table S1: Analytical techniques, instrumentation, and standard methods used for portfolio characterization; Table S2: Global summary statistics of the industrial polypropylene portfolio; Table S3: Summary statistics by polypropylene family; Table S4: Domain population statistics; Table S5: Specification-width statistics for the molecular descriptors defining each product domain; Table S6: Average mechanical, thermal, and optical properties of the identified product domains; Table S7: Literature-supported molecular design rules used to interpret the industrial polypropylene portfolio; Table S8: Benchmarking of the proposed domain framework against international polypropylene portfolios; Table S9: Anonymized grade-level structure–property matrix used for chemometric analyses. Commercial polypropylene grade names were replaced by coded identifiers from PP-001 to PP-081 to preserve industrial confidentiality. The dataset includes the family label, product-domain label, and the molecular, compositional, mechanical, thermal, and optical descriptors used for PCA, PLS-DA, VIP analysis, Pearson correlation analysis, and domain-level profiling.
