# Supplementary material for: Chemometric Organization and Structure–Property Relationships in an Industrial Polypropylene Product Portfolio
Source: Polymers (Basel). 2026 Aug 18;18(16):2009. doi: 10.3390/polym18162009 (PMC13516748; doi:10.3390/polym18162009)
Supplement: Supplementary file 1 [file polymers-18-02009-s001.zip › polymers-4419414-supplementary.pdf]

**Supplementary Information**

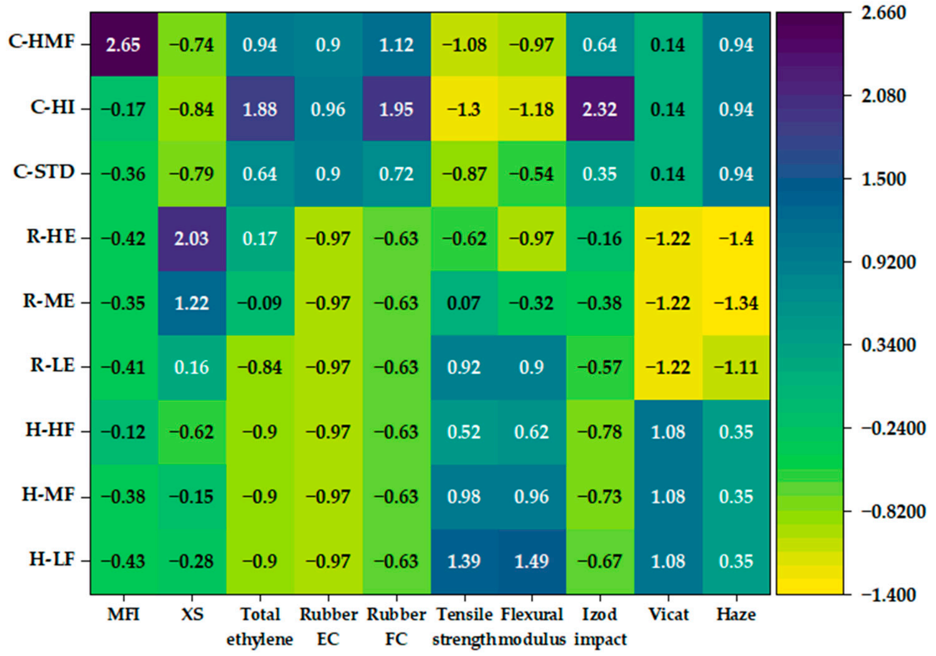


**Figure S1.** Domain-level structure–property heatmap of the industrial polypropylene portfolio. Values correspond to standardized domain means calculated for molecular/compositional, phase-composition, mechanical, thermal, and optical descriptors. Positive values indicate domain means above the portfolio average, whereas negative values indicate domain means below the portfolio average. Domains with small sample sizes, particularly R-LE and C-HMF, should be interpreted descriptively as specialized portfolio niches rather than statistically broad clusters.

Table S1. Analytical techniques, instrumentation, and standard methods used for portfolio characterization.

| Property | Technique | Instrument | Standard/Method |
|---|---|---|---|
| MFI | Melt Flow Index | Melt Flow Tester | ASTM D1238 |
| XS | Xylene extraction | Gravimetric analysis | Internal industrial method |
| Ethylene (%) | FTIR-ATR | Nicolet iS10 | Internal industrial method |
| EC (%) | FTIR-ATR | Nicolet iS10 | Internal industrial method |
| FC (%) | FTIR-ATR | Nicolet iS10 | Internal industrial method |
| Tensile Strength | Universal testing machine | Instron (o equipo equivalente) | ASTM D638 |

| | | | |
|---|---|---|---|
| Flexural Modulus | Universal testing machine | Instron (o equivalente) | ASTM D790 |
| Izod Impact | Impact tester | Industrial QC equipment | ASTM D256 |
| Vicat | Vicat apparatus | Industrial QC equipment | ASTM D1525 |
| HDT | HDT apparatus | Industrial QC equipment | ASTM D648 |
| Haze | Haze meter | Industrial QC equipment | ASTM D1003 |

Table S2. Global summary statistics of the industrial polypropylene portfolio.

| Variable | Mean | SD | Minimum | Maximum | Unit |
|---|---|---|---|---|---|
| MFI | 13.2 | 24.7 | 1.0 | 155.0 | g/10 min |
| XS | 4.3 | 3.0 | 0.5 | 11.5 | wt.% |
| Ethylene | 4.5 | 4.8 | 0.0 | 16.0 | wt.% |
| EC | 45.4 | 2.8 | 40.0 | 50.0 | wt.% |
| FC | 19.4 | 8.7 | 8.0 | 35.0 | wt.% |
| Tensile Strength | 28.2 | 4.3 | 21.0 | 35.0 | MPa |
| Flexural Modulus | 1220 | 240 | 850 | 1650 | MPa |
| Izod Impact | 127 | 135 | 20 | 500 | J/m |
| Vicat | 145 | 7 | 130 | 155 | °C |
| Haze | 47 | 36 | 8 | 95 | % |

Table S3. Summary statistics by polypropylene family.

| Family | n | Mean MFI | Mean XS | Mean Ethylene | Mean EC | Mean FC |
|---|---|---|---|---|---|---|
| Homopolymer | 38 | 7.1 | 2.9 | 0.0 | 0.0 | 0.0 |
| Random Copolymer | 21 | 5.2 | 7.4 | 3.9 | 0.0 | 0.0 |
| Impact Copolymer | 22 | 24.8 | 1.5 | 10.2 | 45.4 | 21.6 |

Table S4. Domain population statistics.

| Domain | n | Portfolio Share (%) |
|---|---|---|
| H-LF | 10 | 12.3 |
| H-MF | 18 | 22.2 |
| H-HF | 10 | 12.3 |
| R-LE | 3 | 3.7 |
| R-ME | 13 | 16.0 |
| R-HE | 5 | 6.2 |
| C-STD | 13 | 16.0 |
| C-HI | 7 | 8.6 |
| C-HMF | 2 | 2.5 |

Table S5. Specification-width statistics for the molecular descriptors defining each product domain.

| Domain | MFI specification width (%) | XS specification width (%) | Ethylene specification width (%) | EC specification width (%) | FC specification width (%) |
|---|---|---|---|---|---|
| H-LF | 22.842 | 20.654 | — | — | — |
| H-MF | 45.571 | 26.115 | 35.556 | — | — |
| H-HF | 23.030 | 37.831 | — | — | — |
| R-LE | 67.857 | 18.605 | 30.000 | — | — |
| R-ME | 23.828 | 14.018 | 13.176 | — | — |
| R-HE | 24.554 | 18.199 | 6.352 | — | — |
| C-STD | 39.767 | 41.507 | — | 16.632 | 27.055 |
| C-HI | 32.958 | 13.333 | — | 21.709 | 15.668 |
| C-HMF | 20.736 | — | — | 15.556 | 16.841 |

Note: Dash symbols indicate descriptors that are not applicable to the corresponding molecular family or that were not used as defining specification variables for that domain.

Table S6. Average mechanical, thermal, and optical properties of the identified product domains.

| Domain | Tensile strength (MPa) | Flexural modulus (MPa) | Izod impact (J/m) | Vicat (°C) | Haze (%) | Main performance profile |
|---|---|---|---|---|---|---|
| H-LF | 33.78 | 1522.69 | 40.6 | 152 | 70 | High stiffness and strength |
| H-MF | 32.00 | 1400.00 | 32.0 | 152 | 70 | Balanced homopolymer performance |
| H-HF | 30.00 | 1320.00 | 25.0 | 152 | 70 | High processability |
| R-LE | 31.73 | 1384.90 | 54.6 | 135 | 20 | Stiff random copolymer |
| R-ME | 28.00 | 1100.00 | 80.0 | 135 | 12 | Transparency–toughness balance |
| R-HE | 25.00 | 950.00 | 110.0 | 135 | 10 | High transparency and ductility |
| C-STD | 23.91 | 1050.00 | 180.0 | 145 | 90 | Standard impact resistance |

| Domain | Tensile strength (MPa) | Flexural modulus (MPa) | Izod impact (J/m) | Vicat (°C) | Haze (%) | Main performance profile |
|---|---|---|---|---|---|---|
| C-HI | 22.00 | 900.00 | 450.0 | 145 | 90 | High toughness |
| C-HMF | 23.00 | 950.00 | 220.0 | 145 | 90 | Impact resistance and high-flow processability |

Table S7. Literature-supported molecular design rules used to interpret the industrial polypropylene portfolio.

| Molecular design variable | Structural effect | Main property consequence | Principal domains affected | Interpretation within this work |
|---|---|---|---|---|
| Increase in MFI | Decrease in molecular weight and melt viscosity | Higher processability, usually lower melt strength and reduced stiffness at very high flow | H-LF, H-MF, H-HF, C-HMF | Explains the differentiation of homopolymer domains and the specialized high-flow character of C-HMF. |
| Increase in ethylene content | Disruption of isotactic propylene sequence regularity and reduction in crystalline perfection | Improved transparency and ductility, usually with reduced stiffness | R-LE, R-ME, R-HE | Explains the progression from low-ethylene random copolymers to high-clarity, ductility-oriented random copolymer domains. |
| Increase in XS | Higher amorphous fraction and reduced effective crystallinity | Greater ductility or optical modification depending on family and morphology | R-ME, R-HE, C domains | Supports the interpretation of XS as a family-dependent descriptor of crystallinity disruption and amorphous-phase contribution. |
| Increase in FC | Higher rubber-phase fraction in heterophasic polypropylene | Higher impact resistance and toughness, with a stiffness penalty | C-STD, C-HI, C-HMF | Explains the separation of impact copolymer domains and the strong association between |

| Molecular design variable | Structural effect | Main property consequence | Principal domains affected | Interpretation within this work |
|---|---|---|---|---|
| | | | | rubber phase and Izod impact. |
| Increase in EC | Higher ethylene content in the elastomeric phase | Improved elastomer efficiency, energy dissipation, and toughness | C-STD, C-HI, C-HMF | Supports the interpretation of EC as a key descriptor of elastomer composition and impact-performance optimization. |
| Crystalline matrix dominance | Higher crystalline polypropylene contribution | Higher tensile strength, flexural modulus, Vicat, and HDT | H-LF, H-MF, H-HF | Explains the stiffness- and thermal-resistance-oriented profile of homopolymer domains. |
| Heterophasic morphology | Polypropylene matrix containing dispersed elastomeric domains | Improved toughness and impact-energy absorption | C-STD, C-HI, C-HMF | Explains the transition from standard-impact to high-impact and high-flow impact copolymer domains. |
| Comonomer-induced crystallinity modification | Smaller or less perfect crystalline structures | Reduced light scattering and improved optical clarity in random copolymers | R-ME, R-HE | Explains why random copolymer domains occupy the transparency–ductility region of the portfolio. |

Table S8. Benchmarking of the proposed domain framework against international polypropylene portfolios.

| Producer/source | Representative grade or family | Reported technical evidence | Relationship with this work | Reference |
|---|---|---|---|---|
| Esenttia | 01H41, low-flow homopolymer | MFI 1.40 g/10 min; tensile strength 34.1 MPa; flexural modulus 1585.8 MPa; Izod 40.6 J/m | Supports H-LF domain: high Mw, high stiffness, moderate impact | Esenttia S.A. |

| Producer/source | Representative grade or family | Reported technical evidence | Relationship with this work | Reference |
|---|---|---|---|---|
| Esenttia | 08H85-1, medium-flow homopolymer | MFI 7.50 g/10 min; tensile strength 31.7 MPa; flexural modulus 1310.0 MPa; Izod 29.4 J/m | Supports H-MF/H-HF transition: MFI increases with stiffness/impact decrease | Esenttia S.A. |
| Esenttia | 01R25, random copolymer | Low-MFI random copolymer with very good impact; recommended for blown film, sheet, strapping, and clarity-demanding applications | Supports R domains: ethylene-modified PP for clarity and impact balance | Esenttia S.A. |
| Borealis | RJ370MO, transparent random copolymer | Tensile strength 27 MPa; tensile modulus 1.05 GPa; visible transmission 90% | Supports R-HE/R-ME domains: reduced crystallinity, transparency, and moderate stiffness | lookpolymers.com |
| LyondellBasell | Impact copolymer family | Described as heterophasic PP with an amorphous phase dispersed in a semi-crystalline PP matrix, giving enhanced impact resistance | Supports C domains: FC/EC-driven heterophasic morphology | lyondellbasell.com |
| LyondellBasell / Basell | Moplen EP2596 impact copolymer | MFR 20 g/10 min; tensile stress 28 MPa; flexural modulus 1450 MPa; Charpy notched impact 9 kJ/m2 | Supports standard/high-flow impact domain behavior | resimport.com.br |
| Basell / Pro-fax | Pro-fax 7523 impact copolymer | Flexural modulus 1220 MPa; Charpy notched impact 10.1 kJ/m2; HDT 76 °C at 0.45 MPa | Supports C-STD/C-HI balance between stiffness and impact | plastore.it |

| Producer/source | Representative grade or family | Reported technical evidence | Relationship with this work | Reference |
|---|---|---|---|---|
| Borealis | PP copolymer portfolio | The product catalog describes copolymers with high stiffness, high impact, and processability for technical applications. | Supports the concept of application-oriented PP product domains | borealisgroup.com |

Table S9. Anonymized grade-level structure–property matrix used for chemometric analyses. Commercial polypropylene grade names were replaced by coded identifiers from PP-001 to PP-081 to preserve industrial confidentiality. The dataset includes the family label, product-domain label, and the molecular, compositional, mechanical, thermal, and optical descriptors used for PCA, PLS-DA, VIP analysis, Pearson correlation analysis, and domain-level profiling

| Grade code | Family code | Family | Product domain | MFI (g/10 min) | XS (wt.%) | Total ethylene descriptor (wt.%) | Rubber EC descriptor (wt.%) | Rubber FC descriptor (wt.%) | Tensile strength (MPa) | Tensile elongation (%) | Flexural modulus (MPa) | Notched Izod impact (J/m) | HDT (°C) | Vicat (°C) | Haze (%) |
|---|---|---|---|---|---|---|---|---|---|---|---|---|---|---|---|
| PP-001 | H | Homopolymer | H-LF | 1.30 | 3.50 | 0.00 | 0.00 | 0.00 | 34.10 | 8.10 | 1585.80 | 40.60 | 100.00 | 152.00 | 70.00 |
| PP-002 | H | Homopolymer | H-LF | 0.50 | 3.50 | 0.00 | 0.00 | 0.00 | 34.00 | 8.00 | 1550.00 | 42.00 | 100.00 | 152.00 | 70.00 |
| PP-003 | H | Homopolymer | H-LF | 2.60 | 2.60 | 0.00 | 0.00 | 0.00 | 34.00 | 8.00 | 1550.00 | 42.00 | 100.00 | 152.00 | 70.00 |
| PP-004 | H | Homopolymer | H-LF | 2.00 | 2.90 | 0.00 | 0.00 | 0.00 | 34.00 | 8.00 | 1550.00 | 42.00 | 100.00 | 152.00 | 70.00 |
| PP-005 | H | Homopolymer | H-MF | 3.20 | 2.35 | 0.00 | 0.00 | 0.00 | 32.00 | 11.00 | 1400.00 | 32.00 | 100.00 | 152.00 | 70.00 |
| PP-006 | H | Homopolymer | H-MF | 3.25 | 2.60 | 0.00 | 0.00 | 0.00 | 32.00 | 11.00 | 1400.00 | 32.00 | 100.00 | 152.00 | 70.00 |
| PP-007 | H | Homopolymer | H-MF | 3.50 | 2.00 | 0.00 | 0.00 | 0.00 | 32.00 | 11.00 | 1400.00 | 32.00 | 100.00 | 152.00 | 70.00 |
| PP-008 | H | Homopolymer | H-MF | 3.50 | 1.25 | 0.00 | 0.00 | 0.00 | 32.00 | 11.00 | 1400.00 | 32.00 | 100.00 | 152.00 | 70.00 |
| PP-009 | H | Homopolymer | H-MF | 3.00 | 3.80 | 0.00 | 0.00 | 0.00 | 32.00 | 11.00 | 1400.00 | 32.00 | 100.00 | 152.00 | 70.00 |
| PP-010 | H | Homopolymer | H-MF | 3.00 | 3.80 | 0.00 | 0.00 | 0.00 | 32.00 | 11.00 | 1400.00 | 32.00 | 100.00 | 152.00 | 70.00 |
| PP-011 | H | Homopolymer | H-MF | 3.00 | 4.10 | 0.00 | 0.00 | 0.00 | 32.00 | 11.00 | 1400.00 | 32.00 | 100.00 | 152.00 | 70.00 |
| PP-012 | H | Homopolymer | H-MF | 3.40 | 5.20 | 0.00 | 0.00 | 0.00 | 32.00 | 11.00 | 1400.00 | 32.00 | 100.00 | 152.00 | 70.00 |

| | | | | | | | | | | | | | | | |
|---|---|---|---|---|---|---|---|---|---|---|---|---|---|---|---|
| PP-013 | H | Homopolymer | H-MF | 3.00 | 3.80 | 0.00 | 0.00 | 0.00 | 32.00 | 11.00 | 1400.00 | 32.00 | 100.00 | 152.00 | 70.00 |
| PP-014 | H | Homopolymer | H-MF | 3.90 | 2.60 | 0.00 | 0.00 | 0.00 | 32.00 | 11.00 | 1400.00 | 32.00 | 100.00 | 152.00 | 70.00 |
| PP-015 | H | Homopolymer | H-MF | 4.60 | 5.10 | 0.00 | 0.00 | 0.00 | 32.00 | 11.00 | 1400.00 | 32.00 | 100.00 | 152.00 | 70.00 |
| PP-016 | H | Homopolymer | H-MF | 8.00 | 1.00 | 0.00 | 0.00 | 0.00 | 32.00 | 11.00 | 1400.00 | 32.00 | 100.00 | 152.00 | 70.00 |
| PP-017 | H | Homopolymer | H-LF | 2.10 | 3.50 | 0.00 | 0.00 | 0.00 | 31.70 | 11.00 | 1241.10 | 29.40 | 100.00 | 152.00 | 70.00 |
| PP-018 | H | Homopolymer | H-MF | 9.50 | 4.00 | 0.00 | 0.00 | 0.00 | 32.00 | 11.00 | 1400.00 | 32.00 | 100.00 | 152.00 | 70.00 |
| PP-019 | H | Homopolymer | H-MF | 8.00 | 4.40 | 0.00 | 0.00 | 0.00 | 32.00 | 11.00 | 1400.00 | 32.00 | 100.00 | 152.00 | 70.00 |
| PP-020 | H | Homopolymer | H-MF | 8.20 | 4.00 | 0.00 | 0.00 | 0.00 | 32.00 | 11.00 | 1400.00 | 32.00 | 100.00 | 152.00 | 70.00 |
| PP-021 | H | Homopolymer | H-HF | 12.50 | 2.40 | 0.00 | 0.00 | 0.00 | 30.00 | 12.00 | 1320.00 | 25.00 | 100.00 | 152.00 | 70.00 |
| PP-022 | H | Homopolymer | H-HF | 12.00 | 2.80 | 0.00 | 0.00 | 0.00 | 30.00 | 12.00 | 1320.00 | 25.00 | 100.00 | 152.00 | 70.00 |
| PP-023 | H | Homopolymer | H-HF | 12.50 | 2.40 | 0.00 | 0.00 | 0.00 | 30.00 | 12.00 | 1320.00 | 25.00 | 100.00 | 152.00 | 70.00 |
| PP-024 | H | Homopolymer | H-HF | 12.50 | 1.00 | 0.00 | 0.00 | 0.00 | 30.00 | 12.00 | 1320.00 | 25.00 | 100.00 | 152.00 | 70.00 |
| PP-025 | H | Homopolymer | H-HF | 12.00 | 2.70 | 0.00 | 0.00 | 0.00 | 30.00 | 12.00 | 1320.00 | 25.00 | 100.00 | 152.00 | 70.00 |
| PP-026 | H | Homopolymer | H-MF | 3.00 | 3.70 | 0.00 | 0.00 | 0.00 | 32.00 | 11.00 | 1400.00 | 32.00 | 100.00 | 152.00 | 70.00 |
| PP-027 | H | Homopolymer | H-MF | 3.00 | 3.30 | 0.00 | 0.00 | 0.00 | 32.00 | 11.00 | 1400.00 | 32.00 | 100.00 | 152.00 | 70.00 |
| PP-028 | H | Homopolymer | H-HF | 17.00 | 2.70 | 0.00 | 0.00 | 0.00 | 30.00 | 12.00 | 1320.00 | 25.00 | 100.00 | 152.00 | 70.00 |
| PP-029 | H | Homopolymer | H-MF | 5.00 | 3.50 | 0.00 | 0.00 | 0.00 | 32.00 | 11.00 | 1400.00 | 32.00 | 100.00 | 152.00 | 70.00 |
| PP-030 | H | Homopolymer | H-HF | 22.00 | 1.00 | 0.00 | 0.00 | 0.00 | 30.00 | 12.00 | 1320.00 | 25.00 | 100.00 | 152.00 | 70.00 |
| PP-031 | H | Homopolymer | H-LF | 2.70 | 2.80 | 0.00 | 0.00 | 0.00 | 34.00 | 8.00 | 1550.00 | 42.00 | 100.00 | 152.00 | 70.00 |
| PP-032 | H | Homopolymer | H-LF | 1.80 | 2.60 | 0.00 | 0.00 | 0.00 | 34.00 | 8.00 | 1550.00 | 42.00 | 100.00 | 152.00 | 70.00 |
| PP-033 | H | Homopolymer | H-LF | 2.70 | 2.80 | 0.00 | 0.00 | 0.00 | 34.00 | 8.00 | 1550.00 | 42.00 | 100.00 | 152.00 | 70.00 |
| PP-034 | H | Homopolymer | H-LF | 2.20 | 2.80 | 0.00 | 0.00 | 0.00 | 34.00 | 8.00 | 1550.00 | 42.00 | 100.00 | 152.00 | 70.00 |
| PP-035 | H | Homopolymer | H-HF | 12.50 | 2.70 | 0.00 | 0.00 | 0.00 | 30.00 | 12.00 | 1320.00 | 25.00 | 100.00 | 152.00 | 70.00 |

| | | | | | | | | | | | | | | | |
|---|---|---|---|---|---|---|---|---|---|---|---|---|---|---|---|
| PP-036 | H | Homopolymer | H-LF | 2.70 | 2.80 | 0.00 | 0.00 | 0.00 | 34.00 | 8.00 | 1550.00 | 42.00 | 100.00 | 152.00 | 70.00 |
| PP-037 | H | Homopolymer | H-HF | 31.00 | 1.00 | 0.00 | 0.00 | 0.00 | 30.00 | 12.00 | 1320.00 | 25.00 | 100.00 | 152.00 | 70.00 |
| PP-038 | H | Homopolymer | H-HF | 30.00 | 1.00 | 0.00 | 0.00 | 0.00 | 30.00 | 12.00 | 1320.00 | 25.00 | 100.00 | 152.00 | 70.00 |
| PP-039 | C | Impact Copolymer | C-STD | 18.00 | 2.70 | 0.00 | 0.00 | 0.00 | 24.00 | 12.00 | 1050.00 | 180.00 | 85.00 | 145.00 | 90.00 |
| PP-040 | R | Random Copolymer | R-ME | 0.70 | 8.00 | 4.20 | 0.00 | 0.00 | 28.00 | 16.00 | 1100.00 | 80.00 | 80.00 | 135.00 | 12.00 |
| PP-041 | R | Random Copolymer | R-ME | 0.20 | 7.50 | 4.00 | 0.00 | 0.00 | 28.00 | 16.00 | 1100.00 | 80.00 | 80.00 | 135.00 | 12.00 |
| PP-042 | R | Random Copolymer | R-ME | 1.70 | 7.50 | 4.30 | 0.00 | 0.00 | 28.00 | 16.00 | 1100.00 | 80.00 | 80.00 | 135.00 | 12.00 |
| PP-043 | R | Random Copolymer | R-HE | 1.60 | 9.50 | 5.10 | 0.00 | 0.00 | 25.00 | 20.00 | 950.00 | 110.00 | 80.00 | 135.00 | 10.00 |
| PP-044 | R | Random Copolymer | R-HE | 1.60 | 9.50 | 5.10 | 0.00 | 0.00 | 25.00 | 20.00 | 950.00 | 110.00 | 80.00 | 135.00 | 10.00 |
| PP-045 | R | Random Copolymer | R-ME | 1.60 | 7.50 | 4.30 | 0.00 | 0.00 | 28.00 | 16.00 | 1100.00 | 80.00 | 80.00 | 135.00 | 12.00 |
| PP-046 | R | Random Copolymer | R-LE | 2.80 | 4.30 | 1.00 | 0.00 | 0.00 | 35.20 | 9.00 | 1654.70 | 43.80 | 80.00 | 135.00 | 20.00 |
| PP-047 | R | Random Copolymer | R-HE | 3.50 | 10.00 | 5.60 | 0.00 | 0.00 | 25.00 | 20.00 | 950.00 | 110.00 | 80.00 | 135.00 | 10.00 |
| PP-048 | R | Random Copolymer | R-ME | 3.50 | 8.50 | 4.90 | 0.00 | 0.00 | 28.00 | 16.00 | 1100.00 | 80.00 | 80.00 | 135.00 | 12.00 |
| PP-049 | R | Random Copolymer | R-HE | 3.20 | 10.00 | 5.60 | 0.00 | 0.00 | 25.00 | 20.00 | 950.00 | 110.00 | 80.00 | 135.00 | 10.00 |
| PP-050 | R | Random Copolymer | R-HE | 3.50 | 10.30 | 5.60 | 0.00 | 0.00 | 25.00 | 20.00 | 950.00 | 110.00 | 80.00 | 135.00 | 10.00 |
| PP-051 | R | Random Copolymer | R-ME | 11.00 | 7.20 | 3.70 | 0.00 | 0.00 | 28.00 | 16.00 | 1100.00 | 80.00 | 80.00 | 135.00 | 12.00 |
| PP-052 | R | Random Copolymer | R-LE | 3.50 | | 0.00 | 0.00 | 0.00 | 30.00 | 14.00 | 1250.00 | 60.00 | 80.00 | 135.00 | 20.00 |
| PP-053 | R | Random Copolymer | R-LE | | | 0.00 | 0.00 | 0.00 | 30.00 | 14.00 | 1250.00 | 60.00 | 80.00 | 135.00 | 20.00 |
| PP-054 | R | Random Copolymer | R-ME | 8.00 | 8.50 | 4.50 | 0.00 | 0.00 | 28.00 | 16.00 | 1100.00 | 80.00 | 80.00 | 135.00 | 12.00 |
| PP-055 | R | Random Copolymer | R-ME | 3.00 | 5.90 | 3.40 | 0.00 | 0.00 | 28.00 | 16.00 | 1100.00 | 80.00 | 80.00 | 135.00 | 12.00 |
| PP-056 | R | Random Copolymer | R-ME | 3.00 | 5.80 | 3.50 | 0.00 | 0.00 | 28.00 | 16.00 | 1100.00 | 80.00 | 80.00 | 135.00 | 12.00 |
| PP-057 | R | Random Copolymer | R-ME | 11.50 | 7.00 | 3.70 | 0.00 | 0.00 | 28.00 | 16.00 | 1100.00 | 80.00 | 80.00 | 135.00 | 12.00 |

| | | | | | | | | | | | | | | | |
|---|---|---|---|---|---|---|---|---|---|---|---|---|---|---|---|
| PP-058 | R | Random Copolymer | R-ME | 11.00 | 6.50 | 3.90 | 0.00 | 0.00 | 28.00 | 16.00 | 1100.00 | 80.00 | 80.00 | 135.00 | 12.00 |
| PP-059 | R | Random Copolymer | R-ME | 12.10 | 8.50 | 4.50 | 0.00 | 0.00 | 28.00 | 16.00 | 1100.00 | 80.00 | 80.00 | 135.00 | 12.00 |
| PP-060 | R | Random Copolymer | R-ME | 12.10 | 8.50 | 4.50 | 0.00 | 0.00 | 28.00 | 16.00 | 1100.00 | 80.00 | 80.00 | 135.00 | 12.00 |
| PP-061 | C | Impact Copolymer | C-STD | 0.85 | 1.00 | 12.10 | 55.00 | 22.00 | 24.00 | 12.00 | 1050.00 | 180.00 | 85.00 | 145.00 | 90.00 |
| PP-062 | C | Impact Copolymer | C-STD | 0.33 | 1.00 | 6.33 | 55.00 | 11.50 | 24.00 | 12.00 | 1050.00 | 180.00 | 85.00 | 145.00 | 90.00 |
| PP-063 | C | Impact Copolymer | C-STD | 0.80 | 2.50 | 12.10 | 55.00 | 22.00 | 24.00 | 12.00 | 1050.00 | 180.00 | 85.00 | 145.00 | 90.00 |
| PP-064 | C | Impact Copolymer | C-STD | 3.70 | 1.25 | 9.90 | 55.00 | 18.00 | 22.80 | 9.50 | 1050.00 | 180.00 | 85.00 | 145.00 | 90.00 |
| PP-065 | C | Impact Copolymer | C-STD | 3.37 | 1.25 | 13.20 | 55.00 | 24.00 | 24.00 | 12.00 | 1050.00 | 180.00 | 85.00 | 145.00 | 90.00 |
| PP-066 | C | Impact Copolymer | C-STD | 3.30 | 1.40 | 6.60 | 55.00 | 12.00 | 24.00 | 12.00 | 1050.00 | 180.00 | 85.00 | 145.00 | 90.00 |
| PP-067 | C | Impact Copolymer | C-STD | 3.30 | 1.40 | 6.60 | 55.00 | 12.00 | 24.00 | 12.00 | 1050.00 | 180.00 | 85.00 | 145.00 | 90.00 |
| PP-068 | C | Impact Copolymer | C-STD | 12.00 | 1.25 | 9.60 | 48.00 | 20.00 | 24.00 | 12.00 | 1050.00 | 180.00 | 85.00 | 145.00 | 90.00 |
| PP-069 | C | Impact Copolymer | C-HI | 7.20 | 1.25 | 13.50 | 50.00 | 27.00 | 22.00 | 18.00 | 900.00 | 450.00 | 85.00 | 145.00 | 90.00 |
| PP-070 | C | Impact Copolymer | C-HI | 10.00 | 1.25 | 13.25 | 53.00 | 25.00 | 22.00 | 18.00 | 900.00 | 450.00 | 85.00 | 145.00 | 90.00 |
| PP-071 | C | Impact Copolymer | C-HI | 7.20 | 1.25 | 14.70 | 42.00 | 35.00 | 22.00 | 18.00 | 900.00 | 450.00 | 85.00 | 145.00 | 90.00 |
| PP-072 | C | Impact Copolymer | C-HI | 33.00 | 1.50 | 14.64 | 48.00 | 30.50 | 22.00 | 18.00 | 900.00 | 450.00 | 85.00 | 145.00 | 90.00 |
| PP-073 | C | Impact Copolymer | C-HI | 7.20 | 1.25 | 13.02 | 42.00 | 31.00 | 22.00 | 18.00 | 900.00 | 450.00 | 85.00 | 145.00 | 90.00 |
| PP-074 | C | Impact Copolymer | C-STD | 3.50 | 1.50 | 0.00 | 0.00 | 16.00 | 24.00 | 12.00 | 1050.00 | 180.00 | 85.00 | 145.00 | 90.00 |
| PP-075 | C | Impact Copolymer | C-HI | 7.20 | 1.25 | 14.70 | 42.00 | 35.00 | 22.00 | 18.00 | 900.00 | 450.00 | 85.00 | 145.00 | 90.00 |
| PP-076 | C | Impact Copolymer | C-STD | 3.30 | 1.25 | 6.60 | 55.00 | 12.00 | 24.00 | 12.00 | 1050.00 | 180.00 | 85.00 | 145.00 | 90.00 |
| PP-077 | C | Impact Copolymer | C-STD | 12.00 | 1.25 | 9.36 | 48.00 | 19.50 | 24.00 | 12.00 | 1050.00 | 180.00 | 85.00 | 145.00 | 90.00 |
| PP-078 | C | Impact Copolymer | C-HMF | 115.00 | 1.60 | 9.23 | 45.00 | 20.50 | 23.00 | 10.00 | 950.00 | 220.00 | 85.00 | 145.00 | 90.00 |
| PP-079 | C | Impact Copolymer | C-HI | 33.00 | 1.50 | 14.64 | 48.00 | 30.50 | 22.00 | 18.00 | 900.00 | 450.00 | 85.00 | 145.00 | 90.00 |

| | | | | | | | | | | | | | | | |
|---|---|---|---|---|---|---|---|---|---|---|---|---|---|---|---|
| PP-080 | C | Impact Copolymer | C-STD | 12.00 | 1.25 | 9.36 | 48.00 | 19.50 | 24.00 | 12.00 | 1050.00 | 180.00 | 85.00 | 145.00 | 90.00 |
| PP-081 | C | Impact Copolymer | C-HMF | 195.00 | 1.60 | 9.45 | 45.00 | 21.00 | 23.00 | 10.00 | 950.00 | 220.00 | 85.00 | 145.00 | 90.00 |
